# A global strategy to mitigate the environmental impact of China’s ruminant consumption boom

**DOI:** 10.1038/s41467-018-06381-0

**Published:** 2018-10-08

**Authors:** Yuanyuan Du, Ying Ge, Yuan Ren, Xing Fan, Kaixuan Pan, Linshan Lin, Xu Wu, Yong Min, Laura A. Meyerson, Mikko Heino, Scott X. Chang, Xiaozi Liu, Feng Mao, Guofu Yang, Changhui Peng, Zelong Qu, Jie Chang, Raphael K. Didham

**Affiliations:** 1 College of Life Sciences, Zhejiang University, Hangzhou, 310058 China; 2 Sustainable Development Research Center, Zhejiang University, Hangzhou, 310058 China; 3 School of Economics, Zhejiang University, Hangzhou, 310058 China; 4 Zhejiang Economic Information Center (Zhejiang Center for Climate Change and Low-carbon Development Cooperation), Hangzhou, 310006 China; 5 College of Computer Science, Zhejiang University of Technology, Hangzhou, 310024 China; 6 Natural Resources Science, University of Rhode Island, Woodward Hall, 9 East Alumni Avenue, Kingston, RI 02881 USA; 7 Department of Biology, University of Bergen, PO Box 7803, Bergen, N-5020 Norway; 8 Institute of Oceanography, National Taiwan University, Taipei, 106 Taiwan; 9 Department of Renewable Resource, University of Alberta, Edmonton, T6G 2E3 Alberta Canada; 10 Institute of Economics, Academia Sinica, Taipei, 115 Taiwan; 11 School of Geography, Earth and Environmental Sciences, University of Birmingham, Birmingham, B15 2TT UK; 12 Center of CEF/ESCER, Department of Biological Science, University of Quebec at Montreal, Montreal, H3C 3P8 Canada; 13 School of Biological Sciences, The University of Western Australia, M092, 35 Stirling Highway, Crawley, WA 6009 Australia; 14 CSIRO Land and Water, Centre for Environment and Life Sciences, 147 Underwood Ave, Floreat, WA 6014 Australia

## Abstract

Rising demand for ruminant meat and dairy products in developing countries is expected to double anthropogenic greenhouse gas and ammonia emissions from livestock by 2050. Mitigation strategies are urgently needed to meet demand while minimizing environmental impacts. Here, we develop scenarios for mitigating emissions under local vs global supply policies using data from 308 livestock farms across mainland China, where emissions intensities are ~50% higher than those in developed nations. Intensification of domestic production and globalized expansion through increased trade result in reductions in global emissions by nearly 30% over a business-as-usual scenario, but at the expense of trading partners absorbing the associated negative externalities of environmental degradation. Only adoption of a mixed strategy combining global best-practice in sustainable intensification of domestic production, with increased green-source trading as a short-term coping strategy, can meet 2050 demand while minimizing the local and global environmental footprint of China’s ruminant consumption boom.

## Abstract (web summary)

Rising demand for ruminant meat and dairy products in developing nations drives increasing GHG and ammonia emissions from livestock. Authors show here that only long-term adoption of global best-practice in sustainable intensification buffered by a short-term coping strategy of green-source trading can offer a way forward.

## Introduction

Ruminant production is one of the major contributors to global environmental degradation^1–4^. Beef, mutton, and milk production contributes 80% of total greenhouse gas (GHG) emissions^2^ and 75% of ammonia (NH_3_) emissions^5^ in the livestock sector. Globally, the GHG emissions from ruminant production have caused US$679 billion in damage costs to ecosystems, and US$13 billion in damage costs to human health^2,6,7^. The NH_3_ emissions from ruminant production have substantial negative effects on the local environment, such as atmospheric haze and nitrogen deposition, leading to human health impacts and eutrophication^5,8,9^. For example, in the United States the damage costs of NH_3_ emissions from the production of livestock export products are estimated to be even higher than the net market value of the exported food^10,11^.

The global consumption of ruminant products has been rising dramatically in the past two decades. Half of the global ruminant meat demand and two thirds of global milk demand are predicted to come from developing nations by 2050, especially China and India^1,12^. Without effective action in developing nations, rising demand for ruminant products is likely to push the global environment close to or beyond a sustainable threshold (the planetary boundary: refs. ^13–15^). Given the increasing globalization of trade and environmental damage^16,17^, the mitigation strategies adopted in developing nations will have an important effect on the livelihoods and welfare of both developing and developed nations.

China is arguably the most important new consumer market for ruminant products^18,19^, and consumption is increasing rapidly^20^. However, the burgeoning demand for ruminant products in China has been met with relatively little regard for environmental impacts thus far. Certainly, over the past 3 years, the Chinese government has adopted a range of policies aimed at reducing livestock pollution, but only a few of these have been specifically targeted at ruminant production^21,22^. Similarly, in other developing nations, policy changes to mitigate the environmental impacts of ruminant production have also been slow in coming. For instance, it was not until 2009 that Brazil issued public policies and interventions in beef and soy supply chains to slow Amazon deforestation^23^, and India only recently developed policy on manure management to reduce GHG emissions in the dairy sector^24^. The key problem for developing nations, and for the world, remains the relatively neglected connection between ruminant consumption and environmental degradation.

Here we evaluate policy options for meeting the demand for ruminant products in China, while minimizing local and global GHG and NH_3_ emissions to 2050. We first develop a dynamic model to analyze the GHG (CH_4_, N_2_O, CO_2_) and NH_3_ emissions along ruminant production chains (feed crop planting, primary feed processing, completed feed processing, livestock rearing, and livestock product processing; Supplementary Figs 1, 2; Supplementary Tables 1-10) using both mass balance assessment and life cycle assessment. These assessments are based on a field survey of 308 ruminant farms (beef cattle, dairy cattle, and sheep) across all 31 provinces of mainland China, and extensive literature review. We then develop a series of scenarios for mitigating emissions under a range of local vs global supply policies. After comparing the local and transferred emissions among these scenarios, we find that a mixed strategy combining global best practice in sustainable intensification of domestic production with increased green-source trading with low emission nations can produce the greatest reduction in global emissions over a business-as-usual scenario.

## Results

### Booming consumption of ruminant products

**Fig. 1:**
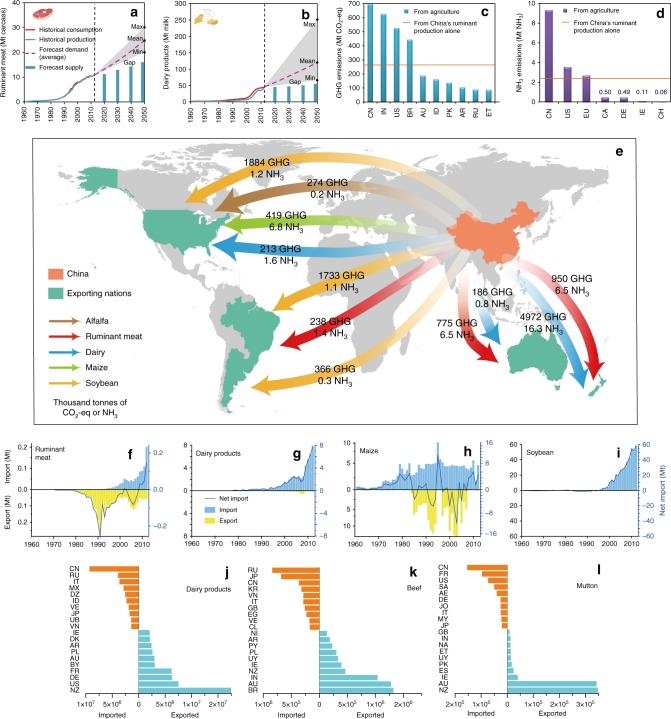
Trends in China’s consumption, production and trade of ruminant products, and the associated local and global transfer of environmental impacts. Ruminant meat and the gaps (**a**), dairy products and the gaps (**b**). Greenhouse gas (GHG) emissions (**c**), and ammonia (NH_3_) emissions (**d**) in domestic ruminant production in China is significantly higher than that of other nations. Moreover China has a large global environmental impact (**e**) through the associated negative externalities of rising net imports of ruminant meat (**f**) and dairy products (**g**) for human consumption, and maize (**h**) and soybean (**i**) for livestock feed from 1961 to 2012. China is now a leading net importer of ruminant products (**j**–**l**, data from 2012, unit: t). Nations used to analyse global consumption trend see Supplementary Table 36. Country codes see Supplementary Table 37. Maps are created in ArcGIS (version 10.1, ESRI)

The consumption of ruminant meat increased exponentially in China from the early 1990s (Fig. 1a) and dairy products increased from the early 2000s (Fig. 1b) when per capita incomes began to rise. As a consequence, the proportion of ruminant meat in total dietary meat consumption (pork, poultry, and ruminant meat) has increased substantially, from 6% to 14%, over the past three decades (Supplementary Fig. 3). Despite the magnitude of the boom, national consumption figures are still lower than global averages (by 2012, China only consumed 14% of world ruminant meat and 7% of dairy products supply, with its ca 20% of global population), and there is significant capacity for further growth (per capita consumption of ruminant meat and milk in China is only 21% and 13%, respectively, of values for the USA in 2012). Combining several methods considering historical consumption patterns and income elasticity (Supplementary Table 11), we project that the demand for ruminant meat and dairy products in China will reach 17.8 and 77.8 kilogram per person per year by 2050; which is still only 48% and 30% of current USA values for ruminant meat and milk consumption, respectively. As a result, the total demand for ruminant products is predicted to double by 2050 (Fig. 1a, b).

### Accelerating emissions from domestic ruminant production

In addition to growing domestic consumption, China has also become one of the largest global producers of ruminant products in its own right. Domestic production has increased 67-fold for ruminant meat and 23-fold for dairy products in past 50 years, exerting great pressure on the environment. In 2012, GHG emissions from ruminant production in China were 270 Tg CO_2_-eq, accounting for 39% of the total domestic agricultural GHG emissions (Fig. 1c). Simultaneously, the domestic NH_3_ emissions from China’s ruminant production alone were 2.3 Mt, accounting for 25% of total domestic agricultural NH_3_ emissions (Fig. 1d). The damage costs from these emissions are high, with NH_3_ emissions from ruminant production in China estimated to cost $21.7 billion to human health and $0.34 billion to ecosystems, while GHG emissions are estimated to cost $0.6 billion to human health and $32.2 billion to ecosystems (Supplementary Tables 12-13). The total damage cost of GHG plus NH_3_ ($54.8 billion) is equal to nearly 50% of the gross annual value of ruminant production in China. Yet, to a great extent, China still cannot meet its domestic demand.

### Transferred emissions to exporting nations

Changing consumption patterns also have a broader global environmental footprint beyond China (Fig. 1e). Despite the large increases in domestic production, China’s consumption of ruminant meat and dairy products far outstrips domestic supply, and demand is increasingly supplemented by overseas imports of both ruminant products and livestock feed (Fig. 1f, i). China has become the world’s largest net importer of dairy products and mutton, and the second largest net importer of beef (Fig. 1j–l). The huge quantity of imported products has transferred tremendous environmental impacts to the rest of the world. In 2012, China’s import of ruminant products and livestock feed transferred 12 Tg CO_2_-eq of GHG emissions and 42.8 Gg NH_3_ emissions to exporting nations (Fig. 1e). Among them, New Zealand received the greatest transferred GHG and NH_3_ emissions, largely through dairy product exports, accounting for 49% and 53% of total transferred emissions, while the USA received 23% of GHG emissions and 23% of NH_3_ emissions, mainly through livestock feed exports (Fig. 1e) (Supplementary Tables 14-15). It is worth emphasizing that, in stark contrast to trade in industrial goods that transfer the impacts from developed nations to developing nations^25^, the impacts from ruminant production are transferring from developing to developed nations.

These current transferred emissions figures are just the tip of the iceberg when projected growth in demand for ruminant products to 2050 is taken into account. According to our projections, China’s domestic production capacity will only meet 66% of ruminant meat demand and 48% of dairy demand by 2050 (Fig. 1a, b). Assuming the supply gap continues to be met by international trade with no change in types of imports under current agreements with exporting countries (business as usual scenario, Supplementary Fig. 4), China will transfer emissions of a staggering 106 Tg CO_2_-eq and 0.65 Tg NH_3_ to the rest of world in 2050 (Supplementary Fig. 5). The sum of domestic and transferred emissions driven by China’s ruminant consumption alone in 2050 would increase GHG to 398 Tg CO_2_-eq, which is equivalent to ~5% of global sustainable thresholds for GHG emissions^26^; and would increase NH_3_ to 2.7 Tg, which is equivalent to ~10% of global sustainable thresholds for NH_3_ emissions^14,27^.

### Local and global mitigation strategies

Under the business-as-usual scenario it is clear that the future environmental costs will be heavy for both developing nations and their trading partners in developed nations. The question is what the best strategy will be to meet growing demand while minimizing both local environmental degradation and the global footprint of impacts across trading partners?

**Fig. 2:**
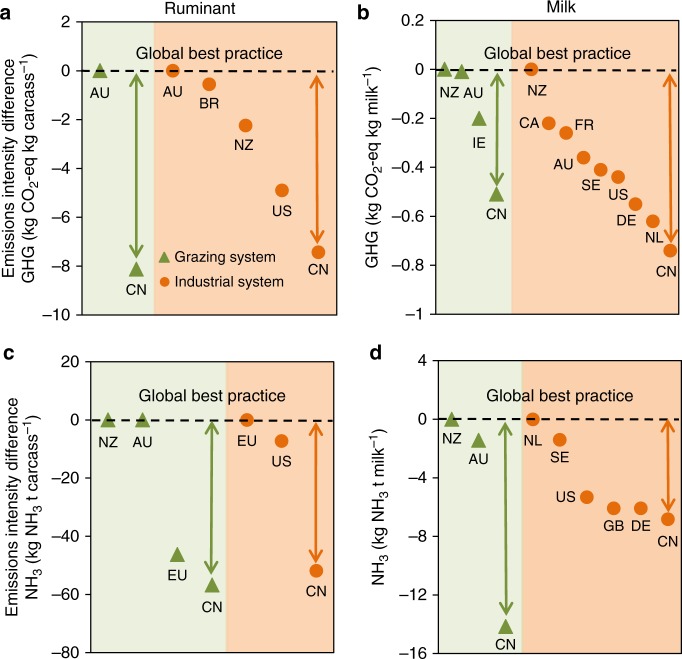
Emissions intensity deficit among the nations. The emissions intensities of GHG (**a**, **b**) and NH_3_ (**c**, **d**) for ruminant meat and milk vary across global production systems (grazing: orange circles; industrial: green triangles). The emissions intensities in China are much higher than global best-practice (the emissions intensity difference in local production efficiency). The data are compiled from literature survey (Supplementary Tables 38-39). For nation codes see Supplementary Table 37

**Fig. 3:**
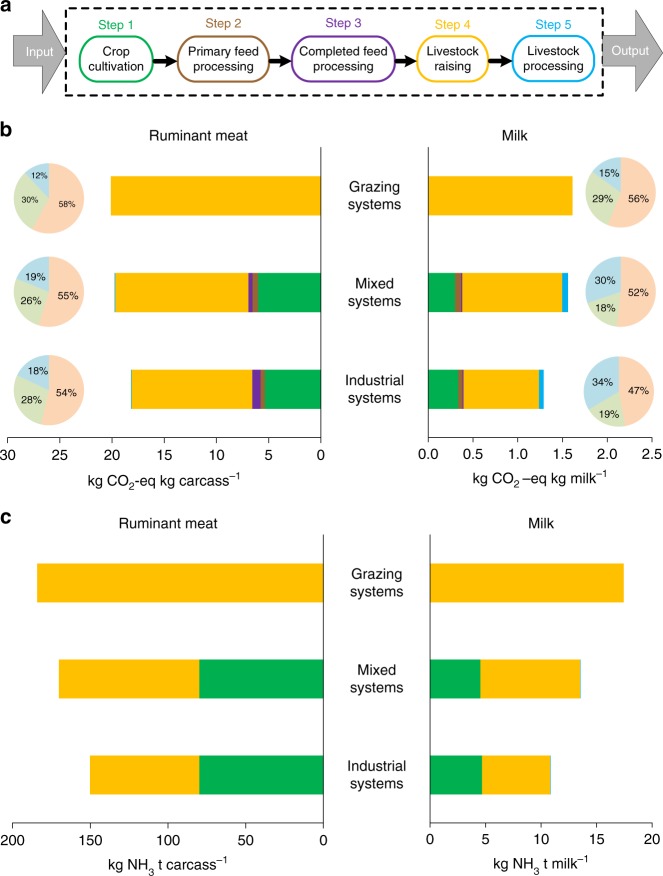
China’s current level of production technology indicated by emissions intensity. The steps of the ruminant production chain (**a**), including crop cultivation, primary feed processing, completed feed processing, livestock raising, and livestock processing. The GHG (**b**) and NH_3_ (**c**) emissions intensities in each of the steps in ruminant meat and milk production. The light blue, yellow, and green in the pie chart in **b** represents CH_4_, N_2_O, and CO_2_ emissions, respectively. The production contribution of each production systems in China see Supplementary Fig. 8, and the diagrams of the calculators see Supplementary Figs 9-11

We carried out a quantitative scenario planning exercise (Supplementary Table 16) in which we used a dynamic systems model to develop six scenarios of supply-side management for mitigating the high emissions predicted under the business-as-usual scenario S0. We considered potential short-term mitigation strategies via increased imports from nations with low emissions intensity in their production systems (which we define as green-source trade), and long-term mitigation via improving domestic technologies toward global best-practice in emissions control (Fig. 2a–d). Central to these mitigation strategies is recognition of the much higher emissions intensities of domestic production in China, compared with leading export nations around the world (Fig. 2a–d). Even for the most efficient of the production systems in China (industrial systems; Fig. 3a, b), GHG emissions intensities are still 27% higher for milk production and 59% higher for beef production than in developed nations (Fig. 2a, b), while NH_3_ emissions intensities in industrial milk and beef systems in China are 43% and 103% higher, respectively, than those in developed nations (Fig. 2c, d).

**Fig. 4:**
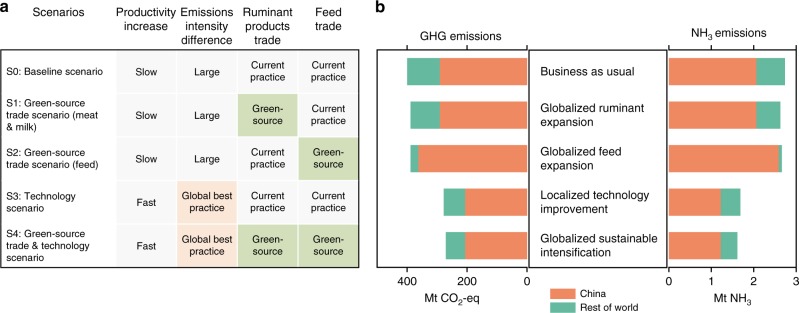
Potential mitigation strategies to meet China’s demand for ruminant products in 2050 and the corresponding domestic and transferred contributions to global greenhouse gases and ammonia emissions. In scenarios S0–S4, the additional demand relative to 2012 is met by either improved technology in domestic production and/or increased imports of ruminant products or feeds. Green-source trade refers to the import of ruminant products or feeds that are produced with global best-practice in emission controls. Current practice means the importing ratios and importing nations are the same as 2012. Two additional endpoint scenarios (total global supply and total domestic supply) are shown in Supplementary Fig. 5 for comparison. For details of scenario description see Supplementary Table 16

In the first scenario S1 (Globalized ruminant expansion) (Fig. 4; Methods), we explore whether strengthening trade can close the supply gap and mitigate emissions in the short term if a green source trade strategy is adopted for importing ruminant products from nations with lower emissions intensities in their ruminant production systems (Fig. 2a–d). We assume the ruminant products gap would be filled solely by increased green source trade, without policies that specifically target domestic production practices. Compared to the reference baseline, this policy would result in small reductions in global GHG by 3% (11 Tg CO_2_-eq) and NH_3_ by 4% (0.1 Tg NH_3_) (Fig. 4), with no emissions changes for China. Transferred GHG would decrease by 11% and NH_3_ by 16%. The reduction in global emissions is due to the lower emissions intensity of ruminant production in trading partners working to global best practice standards (Fig. 2a–d).

Another green-source trade strategy is to import the additional supply of livestock feed required to support domestic intensification from international markets with low emissions production systems. This globalized feed expansion (scenario S2 in Fig. 4) would reduce the global GHG by 3% and NH_3_ by 3% compared with the baseline scenario. The transferred GHG and NH_3_ would decrease by 78% (83 Tg CO_2_-eq) and 92% (0.6 Tg NH_3_), respectively (Fig. 4). At the same time, domestic GHG would increase by 24% (71 Tg CO_2_-eq) and NH_3_ by 25% (0.5 Tg NH_3_) as China shifted to self-sufficient domestic demand for meat and milk. This strategy would have almost the same minor reductions in global NH_3_ emissions as in the scenario for globalized ruminant strategy (S1) (Fig. 4), but a very large decrease in the environmental footprint of impacts on China’s trading partners.

Next, we explore an emissions mitigation scenario in which an increase in ruminant production within China could potentially be achieved by applying the latest global standards in emissions-control technologies (Fig. 4). This localized technology improvement strategy (scenario S3 in Fig. 4) would produce a dramatic step-change reduction in global GHG by 31% (122 Tg CO_2_-eq) and NH_3_ by 39% (1.1 Tg NH_3_), relative to the baseline business-as-usual scenario (Fig. 4). Moreover, if China can close the emissions intensity differences in production (Fig. 2a–d), this would also be a win-win solution for environmental health in China, with domestic GHG reduction of 29% (84 Tg CO_2_-eq) and NH_3_ reduction of 40% (0.84 Tg NH_3_) over baseline. The improvements in technology would also increase productivity and then reduce the gap between the demand and supply of ruminant products. This strategy contributes approximately one third of the net reduction in emissions transfer to global trading partners.

To achieve the transition from current production practices in China to global best-practice, our quantitative life-cycle assessment clearly shows the steps in the production chain that would need to be targeted (Fig. 3). Feed crop planting and livestock rearing steps contribute the major GHG emissions in China (Fig. 3a, b). The high N_2_O emission during feed crop planting is due to large amounts of fertilizer application, accounting for 59% and 58% of the total N_2_O in the full production chain for milk and beef production, respectively. Methane emissions mainly come from enteric fermentation and inefficient manure management during livestock rearing (Fig. 3b), with CH_4_ emissions from manure management accounting for 23% and 26% of the total CH_4_ emissions for milk and beef production, respectively. Similarly, the livestock rearing step in production chains is the major NH_3_ emissions source (51% for beef and 53% for milk, respectively), followed by feed crop planting (47% for beef and 42% for milk) (Fig. 3a, c).

In scenario S4 (Globalized sustainable intensification) (Fig. 4), we combine more rapid adoption of green technology in China with increased green-source trade to close the supply gap, producing comparable domestic benefits to those seen in scenario S3 but additional reductions in global emissions. Under this strategy, the global GHG emissions would decline by 32% (129 Tg CO_2_-eq) and NH_3_ by 41% (1.12 Tg NH_3_) compared with baseline (Fig. 4). At the same time, the transferred environmental impacts to trading partners would also reduce greatly, with GHG declining by 42% (45 Tg CO_2_-eq) and NH_3_ by 43% (0.28 Tg NH_3_), relative to baseline. This scenario produces the most effective combined strategy of long-term improvement in green production technology complemented by improved green-source trade, producing substantial domestic and global emissions reductions.

Finally, we explore two alternative endpoint scenarios that correspond to the extremes of a trade-only scenario S5 (Total global supply; Methods) versus complete localization of supply without supplementation by global trade (at least in the long run) in scenario S6 (Total domestic supply; Methods). We assume that all goods are produced with global best-practice (Supplementary Table 16). The two scenarios result in the largest overall reductions in global GHG (ca 30%) and NH_3_ (ca 40%) (Supplementary Fig. 5), but total domestic supply has a minor advantage simply because of the reduced footprint of product transport. Surprisingly, under the latter scenario, the increase in domestic GHG and NH_3_ relative to the baseline scenario would be minimal (Supplementary Fig. 5), which emphasizes the importance of curtailing the domestic emissions intensity deficit. Of course, the feasibility of operationalizing total domestic supply will be limited by the availability of land to grow feed crops with current technologies, and supply would not meet demand under this scenario.

## Discussion

On balance, the scenarios suggest that local technological improvements across the production chain will be the most effective way to mitigate both domestic and global environmental impacts^28^. Technological change can make a large contribution to global emissions reductions, and China should adopt advanced green technologies through local innovation and technology transfer, such as improved waste recycling to reduce nitrogen pollution^14^, solid–liquid separation to reduce ammonia emission^13,29^ (Supplementary Table 17). Policy instruments such as tax incentives and financial grants could facilitate more rapid adoption of these technologies^30^. For example, recoupling industrial livestock breeding with local feed crop production to alleviate air and wastewater pollution is now supported by some provincial governments (Supplementary Discussion). However, this is still rare in China despite government encouragement (Supplementary Table 18-19), because of high input costs and slow economic return.

Although technological improvement has the greatest potential to mitigate environmental impacts in the long term, it is a slow variable to effect change^31,32^. In terms of productivity, at the national scale, China’s annual increase in milk yield from 1961 to 2013 was only 0.03 t head^−1^ y^−1^ on average. By 2050, if China’s milk productivity were to reach the productivity level that the USA had in 2012, the yield increase rate would need to be sixfold faster, at ca 0.20 t head^−1^ y^−1^. What is worse, ruminant meat productivity in China lags even further behind developed nations and the rate of increase in productivity is very slow (Supplementary Fig. 6). Simultaneously, interim measures will be needed to meet domestic demand in China while reducing environment impacts in the near future.

Compared to technology, trade is a fast variable to effect change. For instance, the quantities of China’s ruminant meat and dairy product imports increased by 70-fold and 29-fold, respectively, in just the past two decades (Supplementary Fig. 6). At least from a local perspective, trade in livestock products could potentially drive rapid improvements in the environment and human health^33,34^. Our scenarios show that both the local and global environmental impacts from China’s consumption of ruminant products could also be mitigated by green-source trade (Fig. 4). Admittedly, of course, there are unquantifiable uncertainties in the transferred emissions, as there is an inherent assumption that key importing nations and importing proportions will remain consistent with the current situation. In the short term the increase in green-source trade could mitigate the current tendency for globalization of big agriculture to encourage unsustainable extractivist approaches to global production at the expense of environmental degradation. Deepening the green in green-source trade may well provide a solution to the long shadow of the ruminant consumption boom in China. However, more careful consideration must be given to the global footprint of trade across the production chain^16,35^. For example, NH_3_ emissions from agricultural production for export have been estimated to cause 5100 premature deaths per year in the USA^10^, while the expansion of the beef cattle industry of Australia has resulted in extensive deforestation (300,000 to 700,000 ha per year), and exacerbated drought impacts in Australia’s natural ecosystems^18^. This suggests that the economic benefits of trade may be weakened if the negative environmental impacts transferred to exporters are too high.

We believe that this mixed strategy combining short-term trade and long-term technological changes also has the highest degree of feasibility in the future. First, the major green-source exporting nations (Fig. 2) have substantial potential to fill the supply gap due to their high self-sufficiency ratios of ruminant products (Supplementary Fig. 7). For example, according to historical data, the self-sufficiency ratio of dairy production has been rising through time, to as high as 250% in USA, 450% in Australia and 9000% in New Zealand (Supplementary Fig. 7). The currently available export amount from just four of the green-sourcing trading partners, Australia, New Zealand, EU, and USA, alone is sufficient to meet 72% of China’s ruminant meat gap and fully meet China’s dairy products gap in 2050 (Supplementary Table 20). Second, exporters have strong enthusiasm for increased trade. For example, the EU recently abolished the 31-year-long milk production quota system, which may promote increased production in EU member nations, and bring more and more new dairy products to Chinese market^36^. Moreover, the overall lifting of the ban on beef imports from Brazil could promote increased trade with China^37^, and this would only be one among a large number of new trade agreements that China has signed over the past decade (Supplementary Table 21). Third, advanced technologies with lower emissions intensity (we define as green technology) have a high potential to be adopted from developed nations^38^, and innovated locally. For example, existing technology for emissions-free livestock housing systems, such as those in the USA^29^, and the pyrolysis of solid manure, such as in the EU^39^, could be widely adopted within China within a relatively short timeframe, and adapted to local conditions.

Of course, the development of ruminant livestock production in China will need more land for forage and would compete with grain crops. However, our estimation shows that the demand for forage could be met by more complete and efficient use of summer fallow croplands and winter fallow croplands, as well as by land use changes policy such as the grain to forage program in China (Supplementary Table 22-23) (Supplementary Discussion). The conversion from forest or grassland to croplands is prohibited in China (Supplementary Table 22), and the gaps in grain feed can be closed through international trade and domestic stock (Supplementary Discussion) (Supplementary Table 24-29). For the environmental impacts, forage production would lead to some increase in GHG and ammonia emissions due to fertilizer use on fallow croplands. For instance, in the mixed strategy, planting additional forage on winter and summer fallow croplands would bring additional GHG and NH_3_ emissions of 18.6 Mt GHG and 0.4 Mt NH_3_, respectively (Supplementary Table 30-31). Finally, substantial alteration of international trade flows of ruminant meat and milk from exporting nations could cause GHG emissions from land use change in the future, especially in those nations that develop agriculture at the expanse of natural ecosystem degradation^23^ (Supplementary Table 32-35). Great caution is needed to avoid or mitigate such effects.

It is also worth pointing out that we intentionally only focus on supply-side mitigation strategies. Of course, changes in demand-side policy have also been widely advocated as mitigation strategies^14,40^, but reducing ruminant consumption in developing nations is not necessarily feasible^35,41^. The estimated 2050 demand for ruminant meat in China that we use in this study (17.8 kg per person per year) is still below the restricted intake designation (18.3 kg per person per year), which has been identified as a global consumption level for reducing the global emissions from livestock below the 2005 level^41^. Moreover, the estimated demand for dairy products in China in 2050 (77.8 kg per person per year) is still lower than the recommended intake of dairy products by the Chinese Nutrition Society (110 kg)^42^, and the world average consumption level (91 kg) ^20^ in 2012. Even the highest estimates of China’s per capita annual demand in 2050, based on the income-dependent dietary choice method of Tilman^28,43^, is only 19 kg of ruminant meat and 187 kg of dairy products, which amounts to just 52% and 79%, respectively, of the current consumption levels in the Western world (Western Europe, Northern America, and Australia in 2009)^20^. As other authors have pointed out^43,44^, reducing environmental impacts through a decrease in ruminant meat consumption might be difficult or infeasible in China and other developing nations at the present time, and we believe that supply-side management is likely to play the fundamental role in mitigating local and global impacts.

Our results clearly show that mitigation of the global environmental impacts of ruminant production must combine a short-term coping strategy of increased green-source trading, with a long-term plan for sustainable intensification using global best-practice in green technology. Reconciling technological improvement and international trade will stimulate synergistic benefits for both human well-being and global environmental sustainability. We believe this integrated strategy is also applicable to other developing nations with underdeveloped technology and rapidly increasing demand. As global consumption of ruminant products is increasing, there is no time to lose.

## Methods

### Forecasting demand for ruminant products in China to 2050

We adopt four methods to produce high vs low projected estimates of future demand. The reason is there is no single universally-accepted method of forecasting rates of change in temporal consumption patterns. In the first method, we use the widely-cited Tilman’s approach^28,43^ to estimate rising consumption patterns from the predictive relationship between per capita GDP increase and dietary choices. Our method uses income-dependent dietary trends to analyze the relationship between per capita GDP and per capita consumption for 94 nations (divided into six economic groups), which account for 90% of the world’s population (the analyzed nations are listed in Supplementary Table 36). In using this approach we implicitly assume that China will change its consumption patterns in a similar manner to that observed elsewhere in the world with increasing income levels. Our results showed that both per capita ruminant meat and milk consumption were significantly related to per capita GDP across economic groups. To estimate future ruminant product demands, we estimated GDP in China by compiling several estimates of projected growth in annual per capita GDP from the literature^28,43–47^. However, this is likely to produce an overestimate of real demand because extrapolation is made on the basis of economic group averages, and not country-specific dietary transitions, when in fact China has always been well below the economic group average for consumption rates.

The second method statistically extrapolates historical trajectories of demand into the future in a business-as-usual manner. We compiled data on historical consumption of ruminant meat (beef, sheep and goat meat) and dairy products (milk excluding butter) from the FAOSTAT database (Global FAO Database; http://faostat.fao.org/), and calculated statistical breakpoints in the relationships (the turning points of changing consumption growth from 1961 to 2009) using the piecewise regression approach presented by Banks-Leite et al.^48^. We then forecasted China’s per capita demand for ruminant meat and dairy products from the reference year (2012) to 2050 using the fitted function for trends beyond the most recent breakpoint. Finally, after obtaining the per capita demand (consumption), we used future predicted human population change data from FAOSTAT to calculate total forecast demand for ruminant products to 2050. This method is likely to produce an underestimate of real demand because the FAO data do not take into account the large missing component of actual consumption that is met by illegal trade in black market ruminant products smuggled into China^49^.

In the third method, we used the Alexandratos’s approach^50^ considering the relationship between consumption and per capita GDP. We compiled data of ruminant meat (beef, sheep, and goat meat) and dairy product (milk, excluding butter) consumption of 103 nations in 2007 from the FAOSTAT database (Global FAO Database; http://faostat.fao.org/). We then forecasted China’s per capita demand for ruminant meat and dairy products from the reference year (2012) to 2050 using the fitted relationship between consumption and per capita GDP. Finally, based on the estimated GDP in China by compiling several estimates of projected growth in annual per capita GDP from the literature^28,43–47^, we obtained the per capita demand (consumption) and the national demand using the future predicted human population change data from FAOSTAT.

Additionally, we adopted Havlík’s approach as the fourth method. The approach was developed according to a food demand forecasting equation based on demand income elasticity, demand price elasticity, price, population, per capita GDP, and historical consumption patterns of ruminant meat and milk^51^. We obtained the historical data from FAOSTAT (e.g., population, price, consumption) and the World Bank (per capita GDP). The per capita GDP projections are the same as those described above. First, we established the regression equations between income elasticities (meat and milk) and per capita GDP using data on 131 countries from the USDA. Second, we projected China’s future income elasticity of meat and milk based on the fitted equations and the forecast per capita GDP. Third, we incorporated the values obtained from these initial steps into Havlík’s equation for food demand forecast to obtain the prior demand quantity for milk and meat^51^. From this we could calculate the optimal demand quantity using future price and price elasticity.

Finally, for the purposes of analysis, we combined these four methods into an average predicted demand to 2050 in order to mitigate potential overestimates or underestimates.

### Forecasting ruminant production to 2050

Again, it is challenging to obtain accurate estimates of rates of change in ruminant production, let alone predict future production. We apply two methods to forecast domestic ruminant production in China.

In the first method, we calculated a statistical extrapolation of historical rates into the future using national-scale data. We compiled data on the national production of ruminant meat (beef, sheep and goat meat) and dairy products (milk, excluding butter) from the FAOSTAT database (Global FAO Database; http://faostat.fao.org/) and calculated statistical breakpoints in the relationships as described above. We then forecast total production to 2050 using the fitted function for trends beyond the most recent breakpoint. However, this is likely to produce an overestimate of real production because linear extrapolation assumes carrying capacity is effectively unlimited (i.e., not asymptotic) and ignores varying biophysical limits within different regions of China.

The second method is based on a more refined province-by-province analysis extrapolating trends in historical production under the assumption of upper limitations on space, water, soil fertility, and other factors in each province. We first projected historical production trends for each province, using data from the Chinese Livestock Statistical Yearbook^52^, and then calculated the sum of the 31 provinces. For the provinces where ruminant meat or dairy production in the last 5 years was stable, we assumed that the current total production would remain stable through time. For other provinces, we assumed the total production would increase, but that production intensity would be constrained by biophysical limits. Therefore we used logistic fitted equations to predict future production in these cases. However, this in turn is likely to represent an underestimate of real future production because it ignores potential technological transitions that could transcend biophysical limits of the natural environment in the future. For the purposes of analysis, we combined these overestimates and underestimates into an average predicted production to 2050.

### Calculation of emissions and environmental impacts

Types and geographical distribution: Ruminant production systems were categorized into three types according to feeding regimes, manure management practices, and available statistical data^22^: grazing systems, industrial systems, and mixed systems. In grazing systems in China, livestock are grazed on extensively managed pastures when the average daily temperature is higher than 10 °C^53^. Most of the excretion is dropped directly onto grassland during the grazing period. The solid part of the excretion is collected when the animals are kept in confinement, but the liquid part is directly leached to the subsoil. These systems are mainly found in grassland areas of Xinjiang, Ningxia, Tibet, and parts of Inner Mongolia (Supplementary Fig. 8).

In industrial systems, livestock rearing occurs in feedlots equipped with an outer cover to maintain stable temperature and humidity regimes, and minimize the extremes of seasonal fluctuations across regions with very different climates. A feedlot is a group of lots or buildings used for the confined feeding, breeding, or holding of animals^54^. This definition includes areas specifically designed for confinement in which manure may accumulate or any area where the concentration of animals is such that a vegetative cover cannot be maintained^54^. A large fraction of the manure produced in industrial systems is discharged into surface waters, with or without some treatment, or dumped into landfills. A part of the solid manure is exported to nearby farms growing vegetables and fruits after a composting treatment. The industrial systems are found in East China where there is high human population density and per capita GDP.

The industrial systems in China are basically landless systems (in a local context) and the feed regime relies on imported feedstock. Feed largely consists of high-quality alfalfa, hay, maize silage, and concentrates^53^. The industrial system is similar to the concentrated animal feeding operations (CAFO) of other nations, such as the USA, but the scale of the industrial system on individual farms in China is lower than typical international CAFO operations^54^.

In mixed systems (i.e., mixed cropping–livestock system), the livestock are raised in both fields and feedlots. The technology level and feed sources are intermediate between the grazing and industrial systems. The solid part of excretion is collected and mainly applied to adjacent cereal crops, whereas the liquid fraction is only partially collected and the remainder is lost by leaching into the subsoil and wider environment. These systems are mainly found in the ecotone between agricultural and grazing areas in China.

System boundary: The physical boundaries of industrial and mixed ruminant production systems are illustrated in Supplementary Fig. 2a. The full system includes five subsystems (steps): feed crop cultivation (step 1), primary feed processing (step 2), completed feed processing to produce complete feed containing all nutrients, essential minerals and vitamins (step 3), livestock rearing (step 4), and raw product processing (step 5). The reason we separate feed processing into two steps (2 and 3) is to facilitate the disentangling of local and transferred environmental impacts: imported feeds are often the primary products (such as maize grain and soybean) and leave environmental impacts of planting and primary processing offsite, whereas the completed feed is mainly processed in local processing plants which causes environmental impacts locally (Supplementary Fig. 2c). The physical boundary of grazing systems is the rangeland that livestock live in.

Calculations of GHG and NH_3_ emissions: To quantify on-farm emissions in China we conducted a field survey of 308 livestock farms representing beef cattle, dairy cattle, sheep, and goats in grazing, industrial, and mixed systems across the 31 provinces of mainland China (Supplementary Fig. 1). The number of selected farms in each province broadly represented the province’s relative contribution to total production in China. The ruminant livestock farms were selected to cover different production systems (industrial, mixed, and grazing systems). We conducted on-farm interviews between 2013 and 2016 and gathered the following information: (a) basic information on livestock farms (location, land size, and herd size; Supplementary Tables 1-3); (b) feed and feeding regime (feed formulation and feed importation), (c) output of livestock products (raw milk yield, live weight of cattle, or sheep for slaughter); (d) manure treatment (how the manure was collected and treated, such as whether or not the farm used solid–liquid separation, or biogas fermentation); and (e) financial support and policy subsidies from the government.

We developed a dynamic model to model China’s beef and milk production and associated emissions across the full production chain for different kinds of production systems based on the STELLA graphic programming system (High Performance Systems, Inc., Version 9.1.2). The model incorporated herd structure, growth stage, and productivity of cattle, feed import ratio, and a range of other factors described in Supplementary Tables 4-10. Then we developed different calculators to evaluate related emissions, including a CH_4_ emissions calculator, N_2_O emissions calculator, CO_2_ emissions calculator, greenhouse gas (GHG) emissions calculator, and ammonia (NH_3_) emissions calculator (Supplementary Figs 9–11). The key input parameters are summarized in Supplementary Tables 4–10. The data for the calculations of nitrogen flux, GHG emissions, and NH_3_ emissions are from an exhaustive literature survey of peer-reviewed publications from Google Scholar, ISI Web of Science, and the China Knowledge Resource Integrated (CNKI) database. The output from ruminant production systems refers only to the processed products (i.e., dairy products refers only to liquid milk, and beef refers to the meat carcass). We conservatively assumed that emissions from beef production are representative of ruminant meat production generally, so we simply considered mutton in the same manner as beef^13^.

Calculation of nitrogen fluxes to the environment: For the entire ruminant production system and each subsystem, we used a mass balance approach to calculate nitrogen fluxes to the environment (*N*_Φ_). The *N*_Φ_ flux includes the emissions of N_2_, NH_3_, NO_*x*_, and N_2_O to the atmosphere and NH_4_^+^-N, NO_3_^−^-N, and ON (organic nitrogen) discharge to water bodies or to soil,

1 $${N_{\Phi (i)}}=N_{{\rm{input}}\Phi (i)} - N_{{\rm{product}}\Phi \left( i \right)} - N_{{\rm{accumulation}}\Phi (i)}$$

2 $$N_{{\rm{input}}\Phi (i)} = N_{{\rm{product}}\left( {i - 1} \right)}$$

where *i* denotes each of the subsystems (steps) of ruminant production; *N*_inputΦ(*i*)_ is N input flux to subsystem *i*; *N*_productΦ(*i*)_ is N product flux from subsystem *i*; *N*_accumulationΦ(*i*)_ is N accumulation in subsystem *i*. For example, in the cropland subsystem the N input includes atmospheric deposition, chemical fertilizer application, biological N fixation, manure application, seed and irrigation; and the product N includes N in harvested crop grain and straw; the accumulated N is the N accumulation in soil. In primary feed processing plants, the N input is from various feed crops (maize, soybean, green maize, and alfalfa), and the N product fluxes include N in primary feed crop products. In completed feed plants, N input is in the form of various feed crop products and feeding additives, and N product fluxes represent the production of a complete feed product (a mixture that meets the nutritional needs of ruminant animals). In livestock feedlots, N input is in the form of complete feed products, and N product fluxes are ruminant products (raw milk and/or live animal). In a livestock product plant, N input is in the form of raw milk or live animal, and N product fluxes are primary ruminant products (liquid milk or carcass). For primary feed plants, completed feed plants, livestock feedlots, and livestock product plants, we assumed that there would be no N accumulated in these subsystems.

Calculation of GHG emissions: The emissions of CH_4_ in ruminant production systems result primarily from enteric fermentation and manure management. The CH_4_ emissions per unit product yield from enteric fermentation ($E_{{\rm{CH}}_{4}{-ef}}$ kg (kg product)^−1^) are calculated as:

3 $$E_{{\mathrm{CH}}_4 - {\mathrm{ef}}}= {\sum}_{j = 1}^m {{\mathrm{EF}}_{{\mathrm{MA}}j} \times N_{j} /Y}$$

where *j* (*j* *=* 1, 2, …, *m*) is the growth stage of ruminant animals (for example, dairy cattle have three growth stages: calf, heifer, and adult cow); EF_MA*j*_ (kg head^−1^ yr^−1^) is the emission factor of CH_4_ from enteric fermentation in stage *j*; *N*_*j*_ is the time-period of stage *j* (yr); *Y* is the yield (kg head^−1^) of a ruminant product during the whole life cycle time of a ruminant animal.

The CH_4_ emissions from manure management ($E_{{\rm{CH}}_{4}{\mathrm{-mm}}}$ kg (kg product)^−1^) are calculated as:

4 $$E_{{\mathrm{CH}}_4 - {\mathrm{mm}}}={\sum}_{j = 1}^m {{\mathrm{EF}}_{{\mathrm{MS}}j} \times N_{j} /Y}$$

where EF_MS*j*_ is the emission factor (kg head^−1^ yr^−1^) of CH_4_ from manure in stage *j*; *N*_*j*_ and *Y* are the same as Eq. (3).

The emissions of N_2_O in ruminant production are from applications of chemical fertilizers and manure in cropland (direct and indirect emissions of N_2_O) and manure management in feedlots. Indirect N_2_O emissions are 1% of the volatilized NH_3_-N and 0.75% of the leached NO_3_-N. The N_2_O emissions from fertilizer application ($E_{{\rm{N}}_2 {\rm{O}}-{\rm{Ca}}}$ kg N_2_O ha^−1^ yr^−1^) are calculated as:

5 $$E_{{\rm{N}}_2{\rm{O}} - {\mathrm{c}}{\rm{a}}}=N_{\rm{in}} \times \left( {{\rm{EF}}_{\rm{ND}} + {\rm{EF}}_{\rm{AD}} \times {\rm{Frac}}_{\rm{GAS} - F} + {\rm{EF}}_{\rm{NL}} \times {\rm{Frac}}_{{\rm{L}} - {\rm{F}}}} \right) \times \frac{{44}}{{28}}$$

where *N*_in_ (kg N ha^−1^ yr^−1^) is the annual amount of fertilizer (or manure) N applied to soils; EF_ND_ is the direct emission factor developed for N_2_O emissions from fertilizer application, kg N_2_O-N (kg of N applied)^−1^ yr^−1^; EF_AD_ is the emission factor developed for N_2_O emissions from atmospheric deposition of N volatilized from managed soil, kg N_2_O-N (kg NH_3_-N + NO_*x*_-N volatilized)^−1^ yr^−1^; Frac_GAS-F_ is the fraction of fertilizer N that volatilizes as NH_3_ and NO_*x*_, %; EF_NL_ is the emission factor developed for N_2_O emissions from leaching/runoff, kg N_2_O-N kg^−1^ yr^−1^; Frac_L–F_ is the fraction of fertilizer N of leaching/runoff, %; 44/28 is the conversion coefficient from N_2_O-N emission to N_2_O emission.

The N_2_O emissions from manure management ($E_{{\rm{N}}_2{\rm{O}} - {\rm{mm}}}$ kg N_2_O ha^−1^ yr^−1^) are calculated as:

6 $$\begin{array}{l}\hskip-20pc E_{{\rm{N}}_2{\rm{O}} - {\rm{mm}}}=\\ \mathop {\sum}\nolimits_{j = 1}^m {\left[ {\left( {{\rm{EF}}_{\rm{NS}} + {\rm{EF}}_{{\rm{AD}} }\times{\rm{Frac}}_{{\rm{GAS}} - M} + {\rm{EF}}_{{\rm{NL}} }\times{\rm{Frac}}_{\mathrm{L - M}}} \right) \times {\rm{NF}}_{j }\times N_j \times \frac{{44}}{{28}}} \right]{\mathrm{/}}Y} \end{array}$$

where EF_NS_ is the direct emission factor developed for N_2_O emissions from manure management, kg N_2_O-N kg^−1^ (of manure N) yr^−1^; Frac_GAS–M_ is the fraction of manure N that volatilizes as NH_3_ and NO_*x*_, %; Frac_L-M_ is the fraction of manure N of leaching/runoff, %; NF_*j*_ is the amount of manure N produced in growth period *j*, kg N head^−1^ yr^−1^.

According to life-cycle analysis, we also calculated the emissions of GHGs (CO_2_, CH_4_ and N_2_O) of pre-production system, including N_2_O and CO_2_ emissions from fertilizer production, CO_2_ emission from transportation and energy consumption.

Calculation of NH_3_ emissions: Considering that NH_3_ has serious environment effects, we calculated it separately. The sources of NH_3_ emissions include fertilizer and manure applications in croplands and manure management in feedlots (including from housing, storage, and spreading). The NH_3_ emissions from croplands ($E_{\mathrm{NH}_3 - {\mathrm c}}$ kg NH_3_ ha^−1^ yr^−1^) are calculated as:

7 $$E_{{\rm{NH}}_3 - {\mathrm {c}}}=N_{\rm{in}} \times {\rm{EF}}_{{\rm{NH}}_3 - {\mathrm {F}}} \times \frac{{17}}{{14}} + N_{\rm{fix}} \times {\rm{EF}}_{{\rm{NH}}_3 - {\mathrm {C}}}$$

where *N*_in_ is the annual amount of fertilizer N applied to soils, kg N ha^−1^ yr^−1^; ${\rm{EF}}_{{\rm{NH}}_3 - {\rm{F}}}$ is the emission factor developed for NH_3_ emissions from fertilizer, kg NH_3_-N kg^−1^ yr^−1^; 17/14 is the conversion coefficient from NH_3_-N emission to NH_3_ emission; *N*_fix_ is the nitrogen fixation rate from nitrogen-fixing crops, kg N ha^−1^ yr^−1^; ${\rm{EF}}_{{\rm{NH}}_3 - {\rm{C}}}$ is the emission factor developed for NH_3_ emissions from nitrogen-fixing crops, 0.01 kg NH_3_ per kg N.

The NH_3_ emissions from manure management in housing ($E_{{\rm{NH}}_3 - {\rm{housing}}}$ kg NH_3_∙ head^−1^ yr^−1^) are calculated as:

8 $$E_{{\rm{NH}}_3 - {\rm{housing}}}=E_{{\rm{housing}} - {\rm{urine}}}{\mathrm{ + }}E_{{\rm{housing}} - {\rm{feces}}}$$

where *E*_housing˗urine_ is the NH_3_ emissions from urine in housing, kg NH_3_ head^−1^ yr^−1^; *E*_housing_feces_ is the NH_3_ emissions of feces in housing, kg NH_3_ head^−1^yr^−1^. *E*_housing_urine_ and *E*_housing_feces_ are calculated as:

9 $$E_{{\rm{housing}} - {\rm{urine}}}=\mathop {\sum}\nolimits_{j = 1}^{m} {\left( {{\rm{TAN}}_{j} \times {\rm{Frac}}_{{\rm{urine}} - j} \times {\rm{EF}}_{{\rm{housing}} - {\rm{urine}} - j}} \right){/}N_{j}}$$

10 $$E_{{\rm{housing}} - {\rm{feces}}}=\mathop {\sum}\nolimits_{j = 1}^m {\left[ {{\rm{TAN}}_j \times \left( {1 - {\rm{Frac}}_{{\rm{NH}}_3 - {\rm{urine}} - j}} \right) \times {\rm{EF}}_{{\rm{housing}} - {\rm{feces}} - j}} \right]{/}N_j}$$

where *N*_*j*_ (yr) indicates the total rearing time of each ruminant animal; TAN_*j*_ is the total amount of NH_4_^+^-N generated from housing in growth stage *j*, kg N head^−1^; Frac_urine–*j*_ is the fraction of urine in manure, %; EF_housing-urine-*j*_ and EF_housing-feces-*j*_ are emission factors of NH_3_ in growth period *j* from urine and feces, respectively, in kg NH_3_ kg^−1^ (of NH_4_^+^-N).

The NH_3_ emissions from manure storage ($E_{{\rm{NH}}_3 - {\rm{storage}}}$, kg NH_3_ head^−1^ yr^−1^) are calculated as:

11 $$E_{{\rm{NH}}_3 - {\rm{storage}}}=E_{{\rm{storage}} - {\rm{urine}}}{\mathrm{ + }}E_{{\rm{storage}} - {\rm{feces}}}$$

where *E*_storage-urine_ is NH_3_ emissions of urine in storage, kg NH_3_ head^−1^ yr^−1^; *E*_storage-feces_ is NH_3_ emissions of feces in storage, kg NH_3_ head^−1^ yr^−1^. *E*_storage-urine_ and *E*_storage-feces_ are calculated as:

12 $$\begin{array}{ccccc}E_{{\rm{storage}} - {\rm{urine}}}=\mathop {\sum}\nolimits_{{\mathrm{j}} = 1}^m {\left( {{\rm{TAN}}_j \times {\rm{Frac}}_{{\rm{urine}} - {{j}}} - E_{{\rm{housing}} - {\rm{urine}} - j}} \right)} \\ \times {\rm{EF}}_{{\rm{storage}} - {\rm{urine}} - j}/N_j\end{array}$$

13 $$\begin{array}{ccccc}E_{{\rm{storage}} - {\rm{feces}}}=\mathop {\sum}\nolimits_{j = 1}^m {\left[ {{\rm{TAN}}_j \times \left( {1 - {\rm{Frac}}_{{\rm{urine}} - j}} \right) - E_{{\rm{housing}} - {\rm{feces}} - j}} \right]} \\ \times {\rm{EF}}_{{\rm{storage}} - {\rm{feces}} - j}/N_j\end{array}$$

where *N*_*j*_ is the rearing time of each ruminant animal; TAN_*j*_ is the total amount of NH_4_^+^-N generated from storage in growth stage *j*, kg N head^−1^; Frac_urine–j_ is the fraction of urine in manure, %; EF_storage-urine-j_, EF_storage-feces-j_ are emission factors of NH_3_ in growth stage *j* from urine and feces, respectively, in kg head^−1^ yr^−1^.

According to life-cycle analysis, we also calculated NH_3_ emissions from pre-production system, including emission from nitrogen fertilizer production and emission from the transportation.

Environmental impacts allocation: If a production or processing process has more than one product, such as maize grain and straw, or soybean cake and soybean oil, or raw milk and cow meat, the environmental impacts produced during the process are allocated to every product based on a certain strategy^1^. In this study, for milk and meat, the environmental impacts are allocated based on their protein amount^1^. For crop, the environmental impacts allocation is based on the economic value fraction^1^ of the target product (concentrate component) and by-products. Since all manure stayed on the farms and was used as fertilizer in the crop production, this was not an output product and therefore no allocation was needed for the manure^2^. The allocation between milk and cow meat is 92% and 8% in this study, respectively.

Calculation of the damage costs of GHG and NH_3_ on human and ecosystem health: We quantified and monetized the effects of GHG and NH_3_ on human and ecosystem health. All three gases (CO_2_, N_2_O, and CH_4_) are important greenhouse gases and their emissions to the atmosphere contribute to climate change, while NH_3_ is an important contributor to the formation of inorganic aerosols^8^. Moreover, atmospheric NH_3_ deposition during rain events^55^, results in acidification of the land and fresh water^56^. We used published coefficients for midpoint environmental impacts to transform emission impacts into endpoint environmental health and human health impacts (see Supplementary Table 13). We measured the damage to human health by disability-adjusted loss of life years (DALY)^57^ and the damage to ecosystem health by biodiversity-adjusted hectare years (BAHY)^7^. A unit of DALY is equivalent to a damage cost of 74,000 EUR_2003_, and a unit of BAHY is equivalent to a damage cost of 1400 EUR2003 (1 = $ current, considering the inflation from 2003 to 2016, www.dollartimes.com/calculators/inflation.htm).

### Scenario analyses

We calculated local and global GHG and NH_3_ emissions across the whole life cycle of the ruminant production process to 2050. We considered a reference scenario (business-as-usual) and six contrasting scenarios of variation in domestic production versus international trade modes (as detailed in Supplementary Table 16). For comparative purposes, we assumed that in all scenarios China’s demand for ruminant products in 2050 was fixed at the levels determined in the forecasting analysis above; i.e. 24.7 Mt for ruminant meat and 107.8 Mt for dairy products. Technology improvements included an increase in productivity and decrease in GHG and NH_3_ emissions intensities. We also assumed that the projected increase in the production of ruminant products relative to 2012 would be realized through industrial production systems. In addition, the Chinese government clearly promoted the grain to feed program in 2015, i.e., changing maize grains to silage or other forage in the cold regions of northern China (Supplementary Discussion). Hence, we assumed that the future expansion of feed fields would predominantly occur in existing farmland instead of forests, so that there would be no large increase in GHG emissions resulting from land use changes. Consequently, in the scenario settings, GHG emissions from land use changes were not a component that was incorporated in this study. For incorporation of dynamic change among competing land uses, see discussion in Supplementary Discussion.

We only considered international trade, while ignoring domestic redistribution among provinces within China. The GHG and NH_3_ emissions are those included in the full production chain and transportation of goods and products, but excluding the emissions from energy used for freezer storage due to lack of data (see parameters in Supplementary Tables 14, 15). In scenario calculation, we also considered the decreasing of emission intensities in major exporting nations due to technology improvement. Based on the time series of CO_2_-eq emissions for beef meat and milk production of 1961–2016 from the FAOSTAT database (Agri-Environmental Indicators-Emission intensities), we estimated the emissions intensities by 2050 (Supplementary Table 32). Due to the lack of historical data on NH_3_ emissions intensities, we assumed that the deceasing ratio of NH_3_ emission intensities of the major exporting nations is the same as that of the GHG emissions. Taking New Zealand as an example, the GHG emissions intensity of milk production in 2050 is projected to decrease by nearly 13%, so the NH_3_ emissions intensity of milk in 2050 is assumed to decrease by 13% (see Supplementary Table 33). For soybean and alfalfa, we assumed that emissions intensities increased by 30% (Supplementary Tables 34-35). We used data on the proportion of importing nations and imports of various types of products in 2012 from FAOSTAT **(**Supplementary Table 15) for future import calculation. All international transportation was assumed to be via shipping, with distance data obtained from Distance Netpas 3.2 (https://netpas.net/products/product_detail_DT_CN.php). The loss ratios during transportation were set to 8% for milk, 5% for ruminant meat, and 3% for livestock feed.

### Life cycle compilation and analysis

The GHG and NH_3_ emissions intensities of beef and milk in exporting nations were collected from literature sources. To find candidate publications, we searched for papers reporting LCAs for soybean, alfalfa, maize, liquid milk and beef meat using Web of Knowledge, Google Scholar, and PubMed. We chose all published LCAs that detailed the system boundaries of the study and that included and delimited the full cradle to product portion of the food/crop lifecycle of potential GHG and NH_3_ emissions, including emissions from pre-farm activities such as fertilizer production, but excluding emissions from land-use change (Supplementary Table 38). If the studies analyzed production from cradle to farm gate, we recalculated the results to our system boundary. For consistency, we chose the studies in which the allocation method was protein allocation (or we recalculated based on the available allocation information provided). In order to better compare the emissions between different food groups, we calculated emissions per kilogram. The functional unit of dairy cattle is liquid fresh milk (protein content 3.2%). The final product of beef cattle is carcass meat (protein content 20%). We recalculated the GHG emissions based on the coefficients of IPCC 2014^58^. Because few data were available on NH_3_ emissions from the full production chain, we recalculated NH_3_ emissions from cattle breeding based on the proportions of each component of total emissions measured across the whole production chain reported by^59^ Thomassen et al. (Supplementary Table 39).

### Uncertainty analysis

Uncertainties associated with the calculated emissions were estimated using a Monte Carlo simulation (10,000 runs). The major uncertainty came from the emission factors collected from the published literature. Another important uncertainty source arises from the activity data used in this study. Input variables related to emission factors, cattle population, productivity of cattle, fertilizer use in feed crop cultivation, and some model parameters have been assumed to vary and have been randomly sampled. The medians and 95 percent confidence intervals for these parameters were calculated and used to characterize the uncertainties associated with the GHG and NH_3_ emissions intensities. The probability distribution functions and uncertainty values assumed for the GHG and NH_3_ emission model parameters and data input variables are summarized in Supplementary Table 30 and Supplementary Table 31 of the Supplementary Information. Taking the industrial milk production system as an example, the Monte Carlo simulation showed that GHG and NH_3_ emissions intensities had an uncertainty range of 0.95–1.84 kg CO_2_-eq per kg milk and 0.0049–0.0167 kg NH_3_ per kg milk, respectively.

## Electronic supplementary material

Supplementary Information

## Data Availability

The authors declare that all other data supporting the findings of this study are available within the article and its Supplementary Information files, or are available from the corresponding author upon reasonable request.
