# Supplementary material for: A global strategy to mitigate the environmental impact of China’s ruminant consumption boom
Source: Nat Commun. 2018 Oct 8;9:4133. doi: 10.1038/s41467-018-06381-0 (PMC6175953; doi:10.1038/s41467-018-06381-0)
Supplement: Supplementary file 1 — Supplementary Information [file 41467_2018_6381_MOESM1_ESM.docx]

**SUPPLEMENTARY INFORMATION**

**A global strategy to mitigate the environmental impact of China’s ruminant consumption boom**

Yuanyuan Du, Ying Ge, Yuan Ren, Xing Fan, Kaixuan Pan, Linshan Lin, Xu Wu, Yong Min, Laura A. Meyerson, Mikko Heino, Scott X. Chang, Xiaozi Liu, Feng Mao, Guofu Yang, Changhui Peng, Zelong Qu, Jie Chang^*^ and Raphael K. Didham^*^

correspondence to: [jchang@zju.edu.cn](mailto:jchang@zju.edu.cn), co-correspondence: [raphael.didham@csiro.au](mailto:raphael.didham@csiro.au)

**Contents:**

Supplementary Figures 1 to 11

Supplementary Tables 1 to 39

Supplementary Discussion

Supplementary References

**Supplementary Figures**


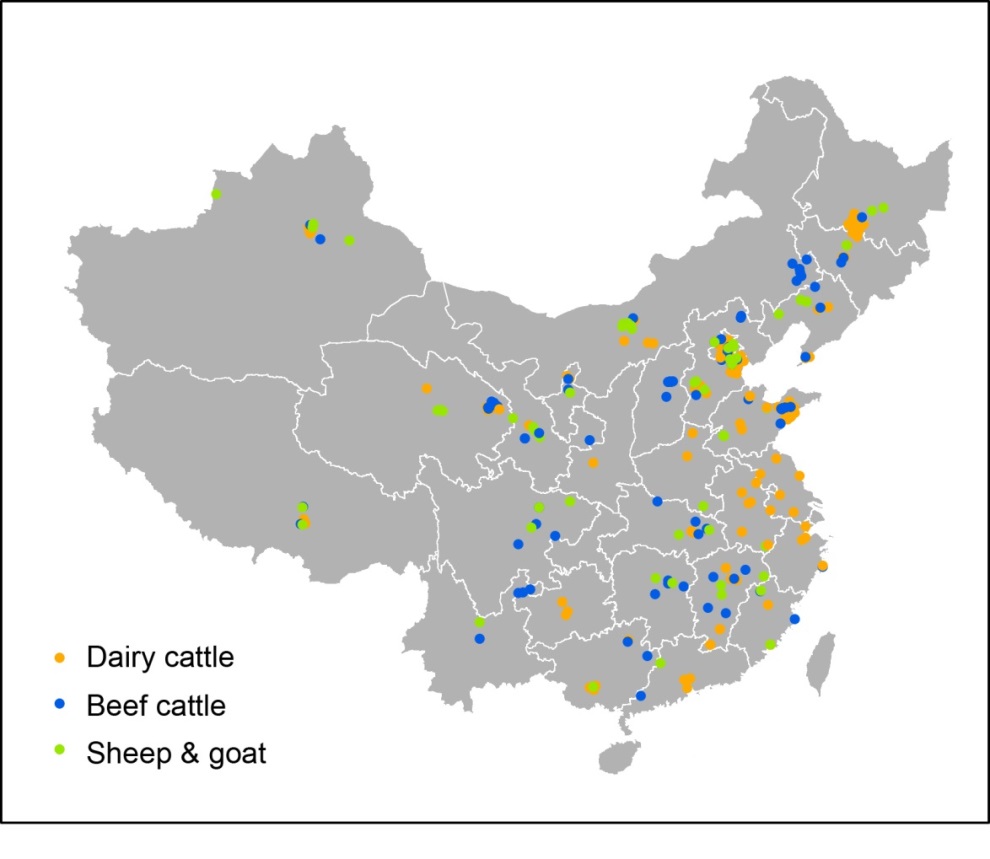


**Supplementary Figure 1 Ruminant livestock farms surveyed in this study.** The intensity of survey effort in each of the 31 provinces of mainland China was proportional to ruminant production per province. There are almost no industrial or mixed ruminant livestock farms in the western part of China, which is dominated by extensive mountain chains (e.g. Qinghai-Tibet Plateau and Yunnan-Guizhou Plateau) and deserts (e.g. Taklimakan Desert, Badanjilin Desert, Mu Us Desert and others). These areas predominantly support extensive grazing systems for sheep, managed by small-holder farmers. Maps are created in ArcGIS (version 10.1, ESRI)


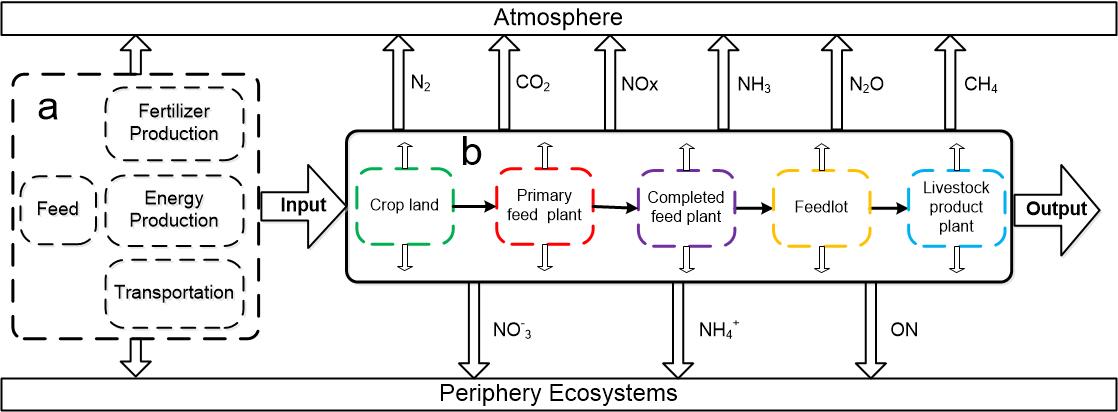


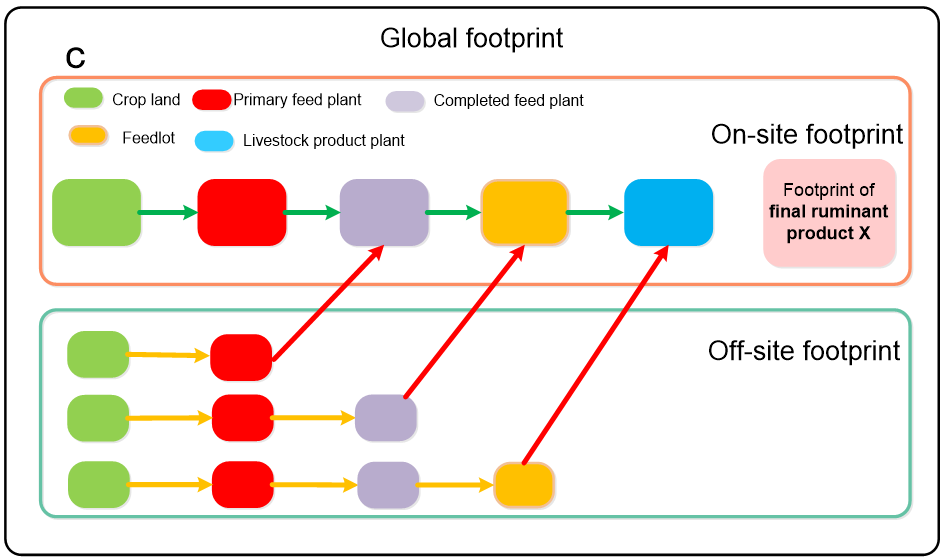


**Supplementary Figure 2 Diagrammatic representation of the physical boundaries of ruminant production systems. a**. Production practices outside the boundary are relevant as inputs to the ruminant production system; **b**. five subsystems within the boundary of the whole production system for industrial or mixed farming system: cropland (Step 1, green box), primary feed processing (Step 2, red box), completed feed processing (Step 3, purple box), feedlot rearing (Step 4, orange box), and processing of livestock products (Step 5, blue box); **c**. the global footprint of emissions from ruminant production systems, combining the on-site and off-site footprint of each of the five steps in the production chain.


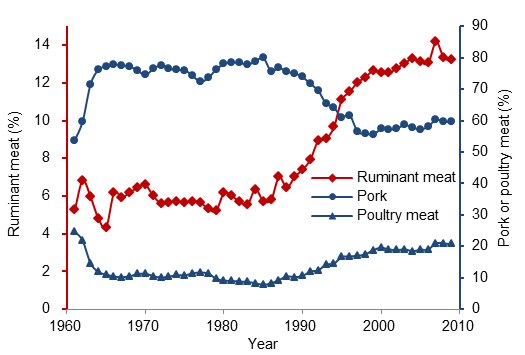


**Supplementary Figure 3 Historical consumption ratio of ruminant meat, pork and poultry as a proportion of total meat from 1961 to 2009 in China.** Data source: FAOSTAT database^3^.

**Supplementary Figure 4 The dominant countries providing imports of ruminant meat, dairy products, maize and soybean to China in 2012.** Data are from FAOSTAT database^3^.


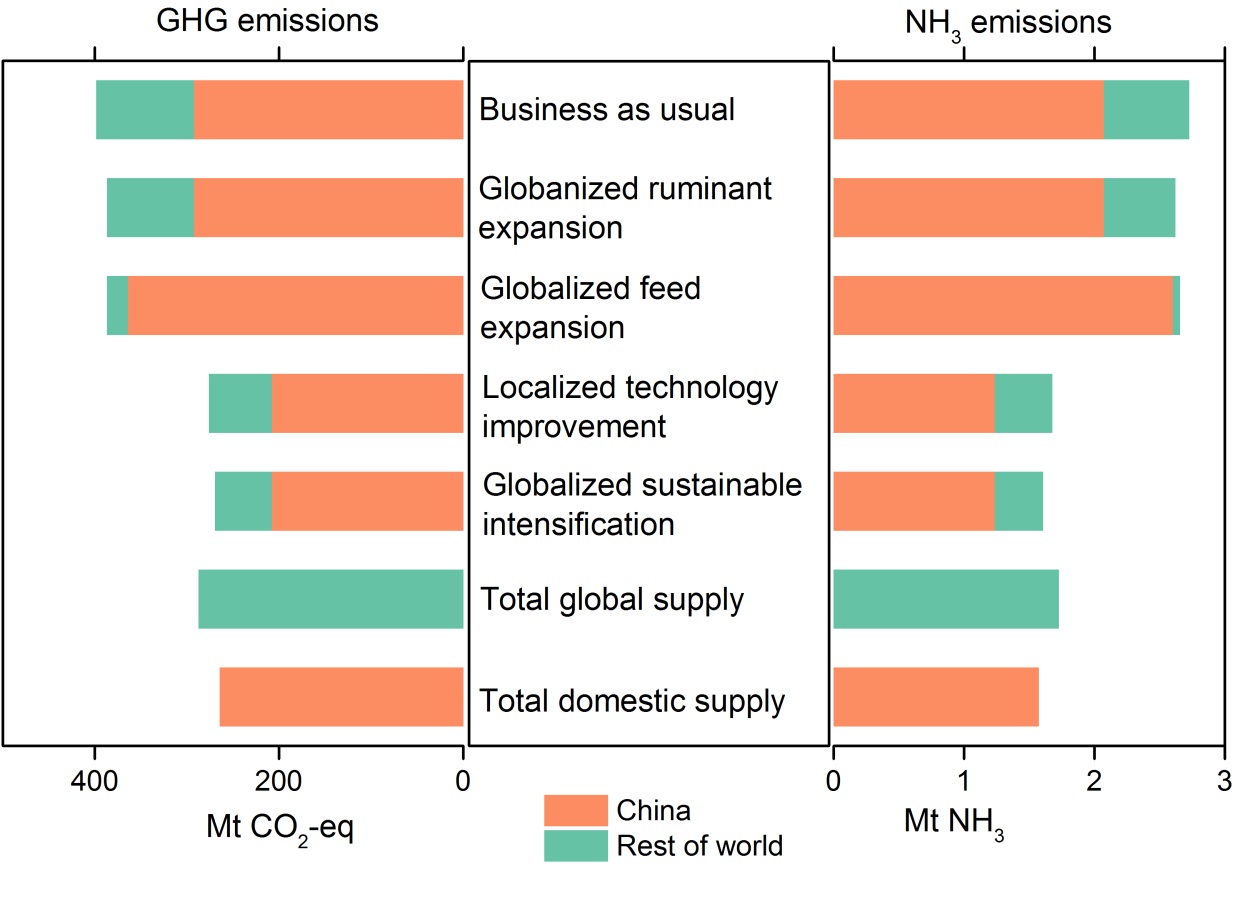


**Supplementary Figure 5 Potential mitigation strategies to meet China’s demand for ruminant products in 2050 and the corresponding domestic and transferred GHG and NH_3_ emissions.** Note the addition of the two endpoint scenarios (Total global supply and Total domestic supply) in comparison to Figure 4.


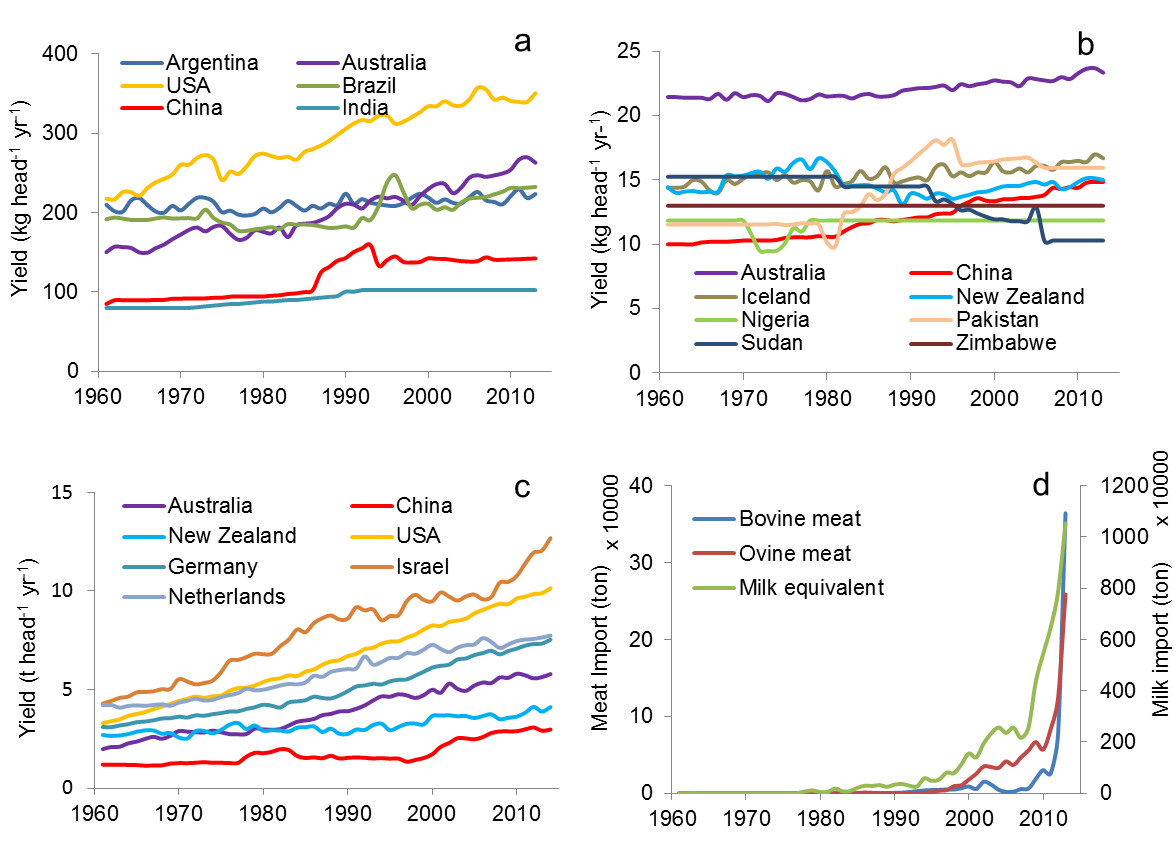


**Supplementary Figure 6 Historical trends for ruminant animal yield of major nations and China’s ruminant product import.** **a**. Beef meat production in the six nations accounting for over 50% of the world’s total production in 2013; **b**. sheep and goat meat production in the six nations accounting for over 50% of the world’s total production in 2013; **c**. milk production in the seven nations accounting for 30% of the world’s total production in 2013; **d**. quantity of imported beef meat (bovine), sheep and goat meat (ovine) and milk in China from 1961 to 2013. Data source: FAOSTAT database^3^.

**
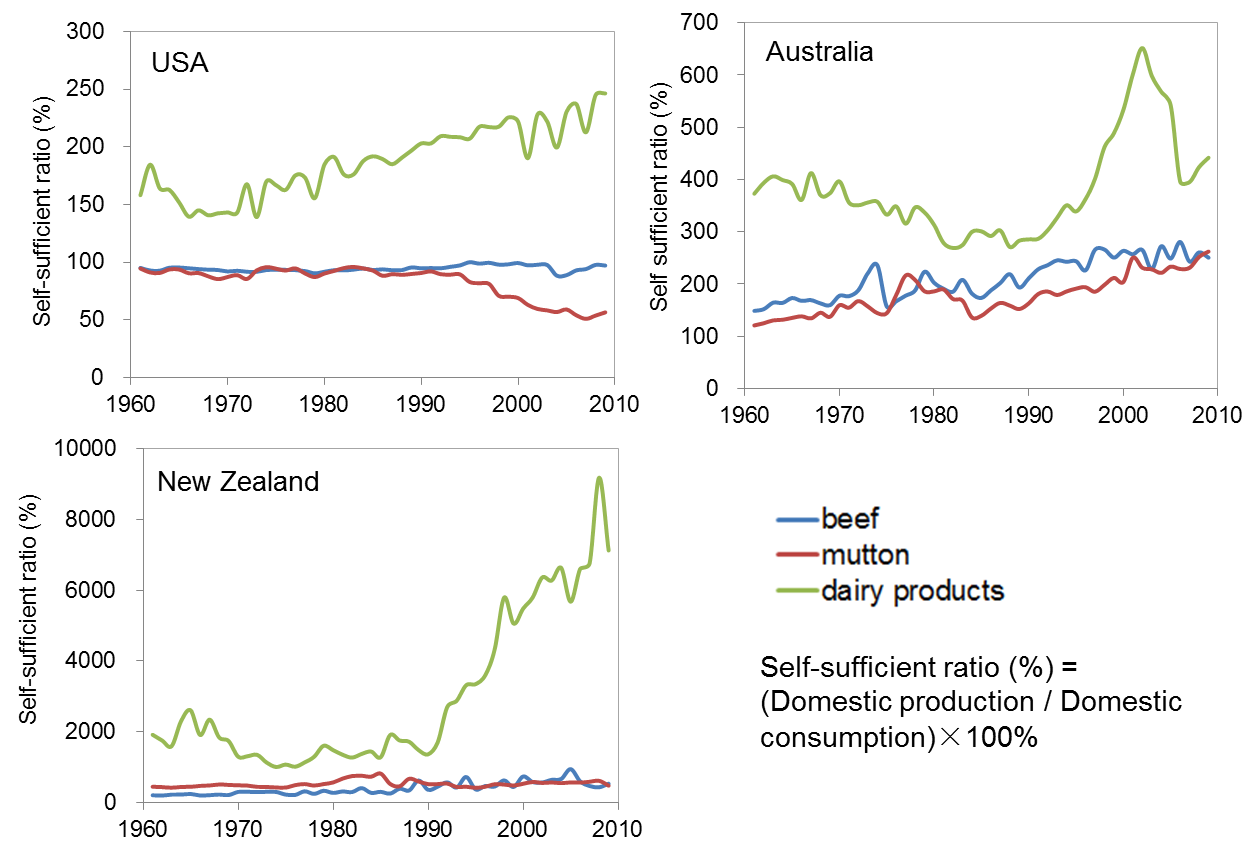
**

**Supplementary Figure 7 Self-sufficient ratios of ruminant products in USA Australia and New Zealand.** Despite the huge damage costs to human health and the environment via the negative externalities of imports (Supplementary Table 12), the major exporting nations continue to increase their exports. Data source: FAOSTAT database^3^.


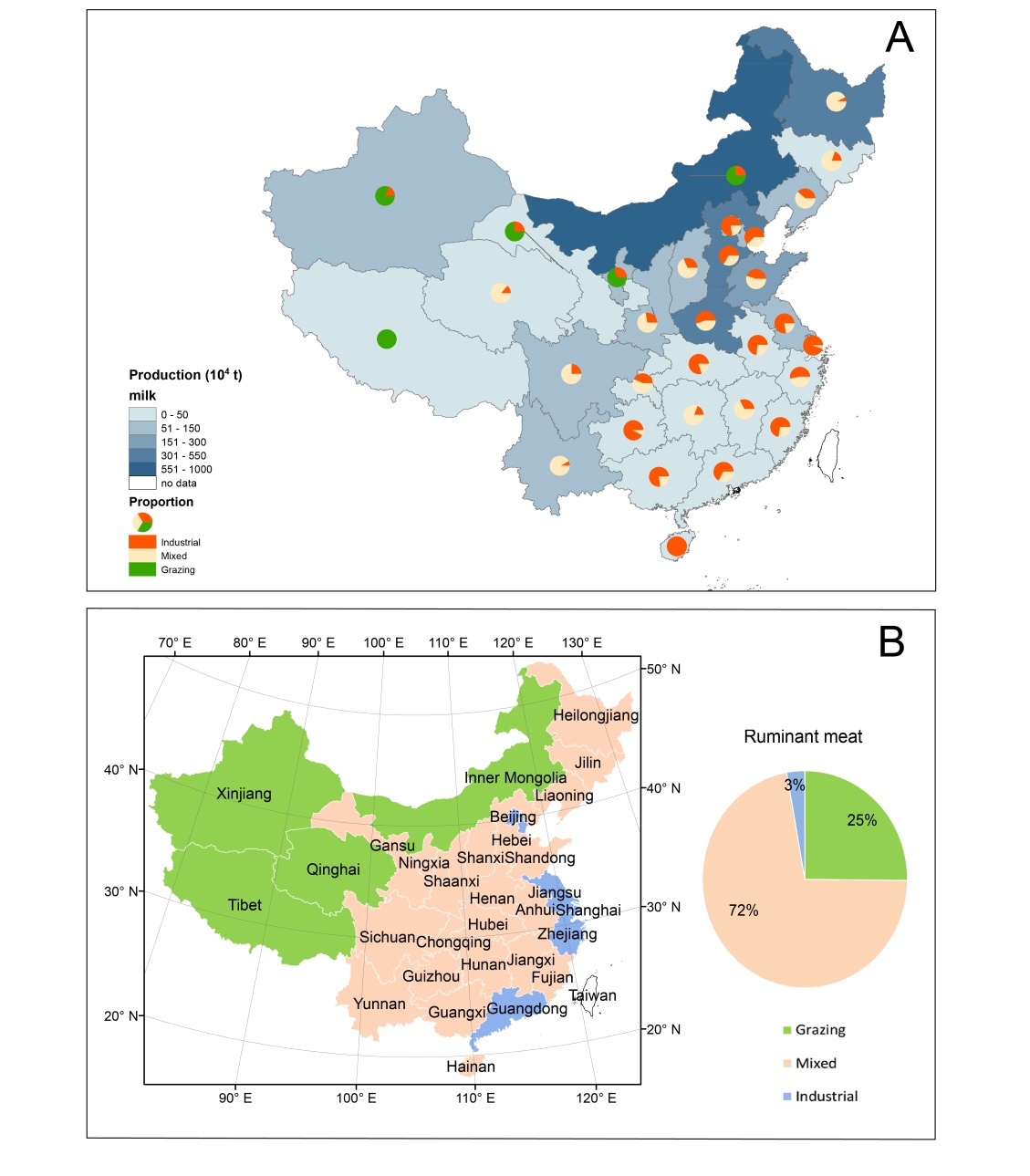


**Supplementary Figure 8 Grazing industrial and mixed ruminant production systems in the 31 provinces of mainland China. a**. Total milk production (colour intensity), and proportion of production (piecharts) from different production systems in each province; **b**. proportion of total meat production in China that is produced in different production systems (piechart), and dominant production type in each province (colour codes). Maps are created in ArcGIS (version 10.1, ESRI)


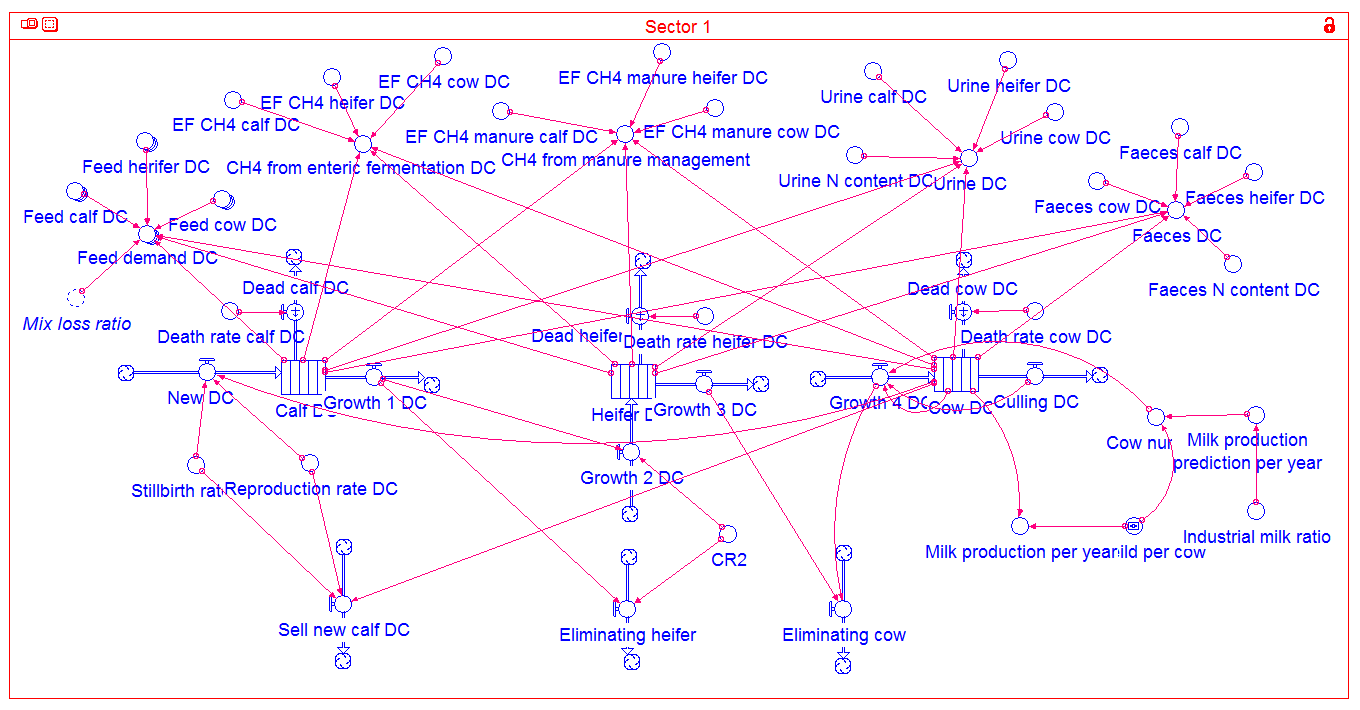


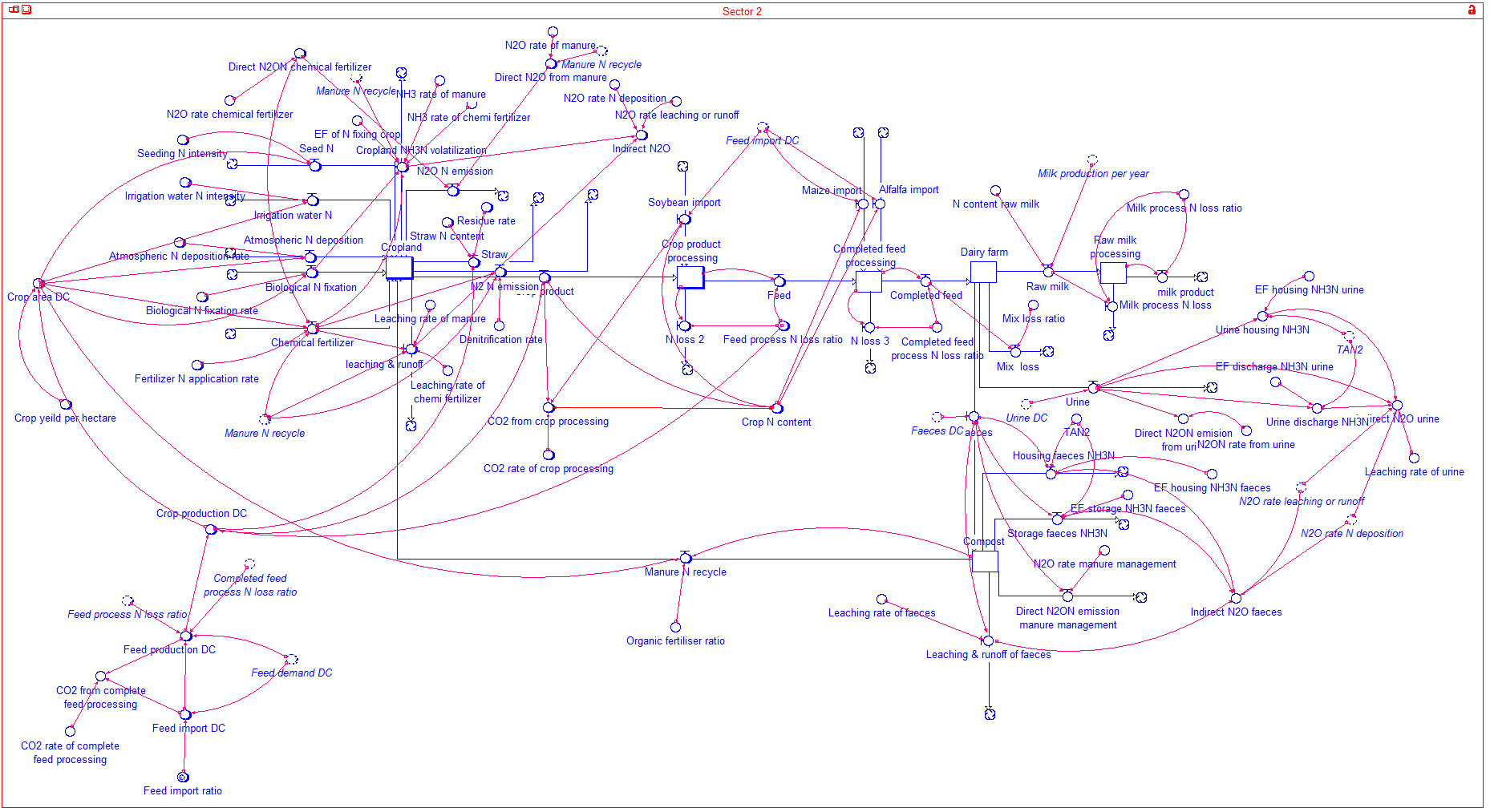


**Supplementary Figure 9 Diagram of the nitrogen flux calculator in STELLA graphic design (taking dairy cattle as an example).** Squares represent stocks of N in feed, cattle, ruminant products, manure, and faeces. Fluxes of materials are represented by thick arrows with attached circles (flows). Circles are auxiliary variables (converters). Thin arrows represent information flows. All the stocks, flows and converters with multilayer structures are arrayed variables (a feed classification array).


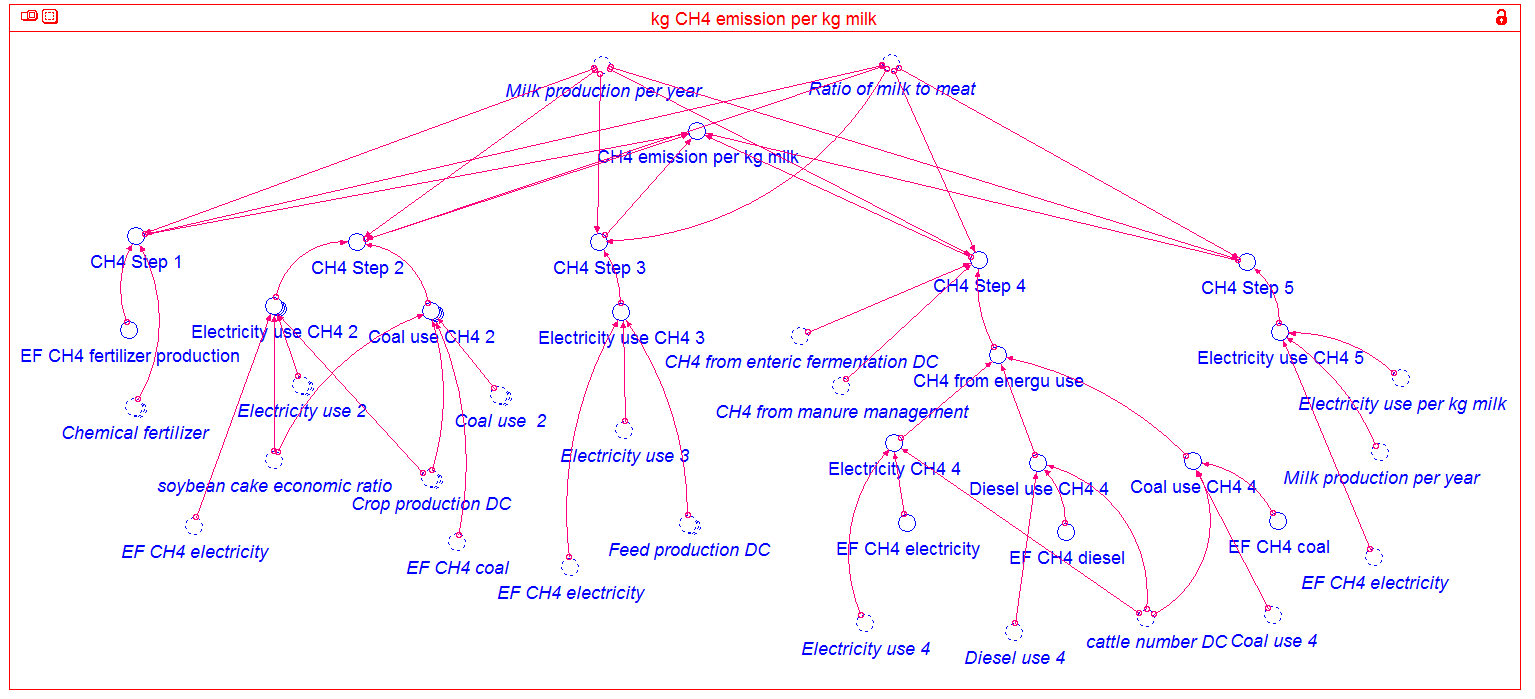


aa


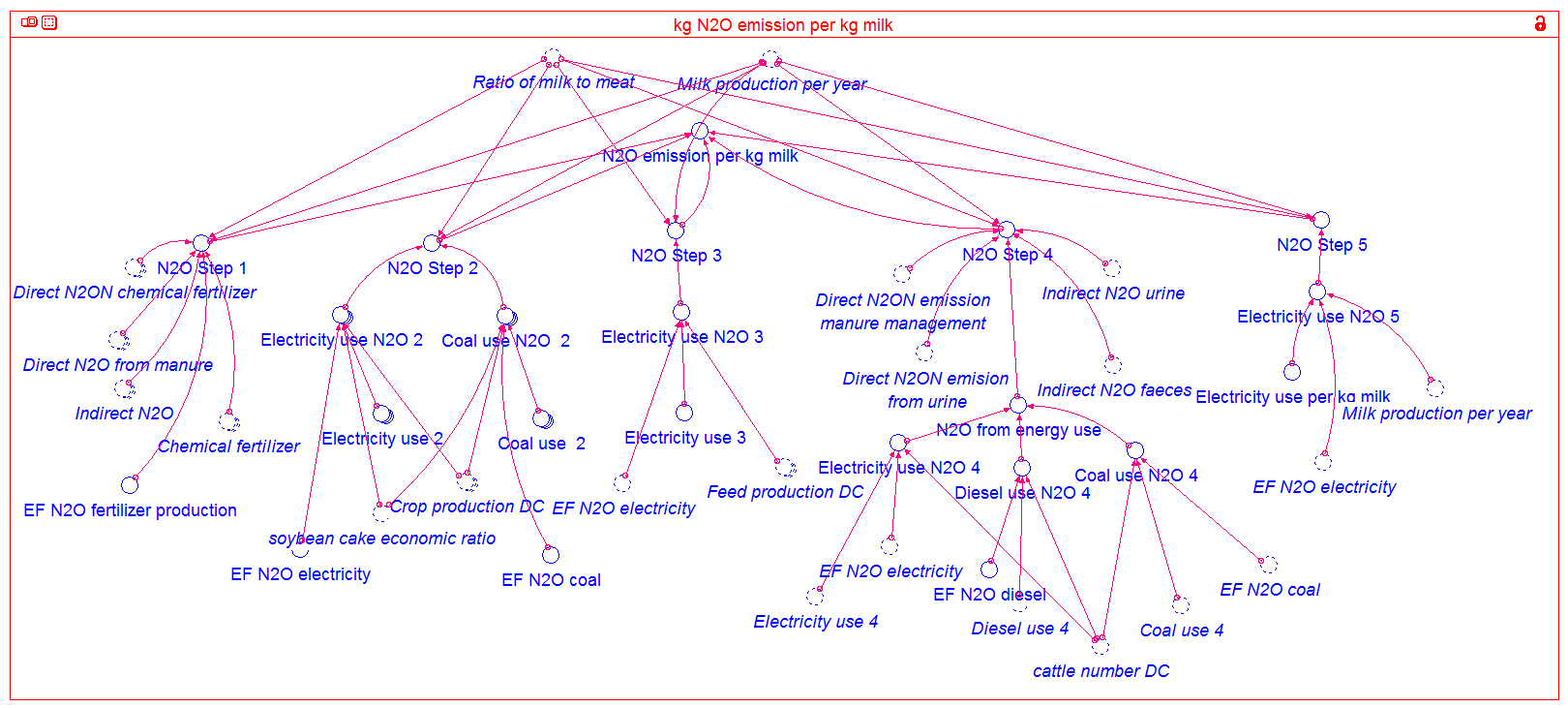


ba


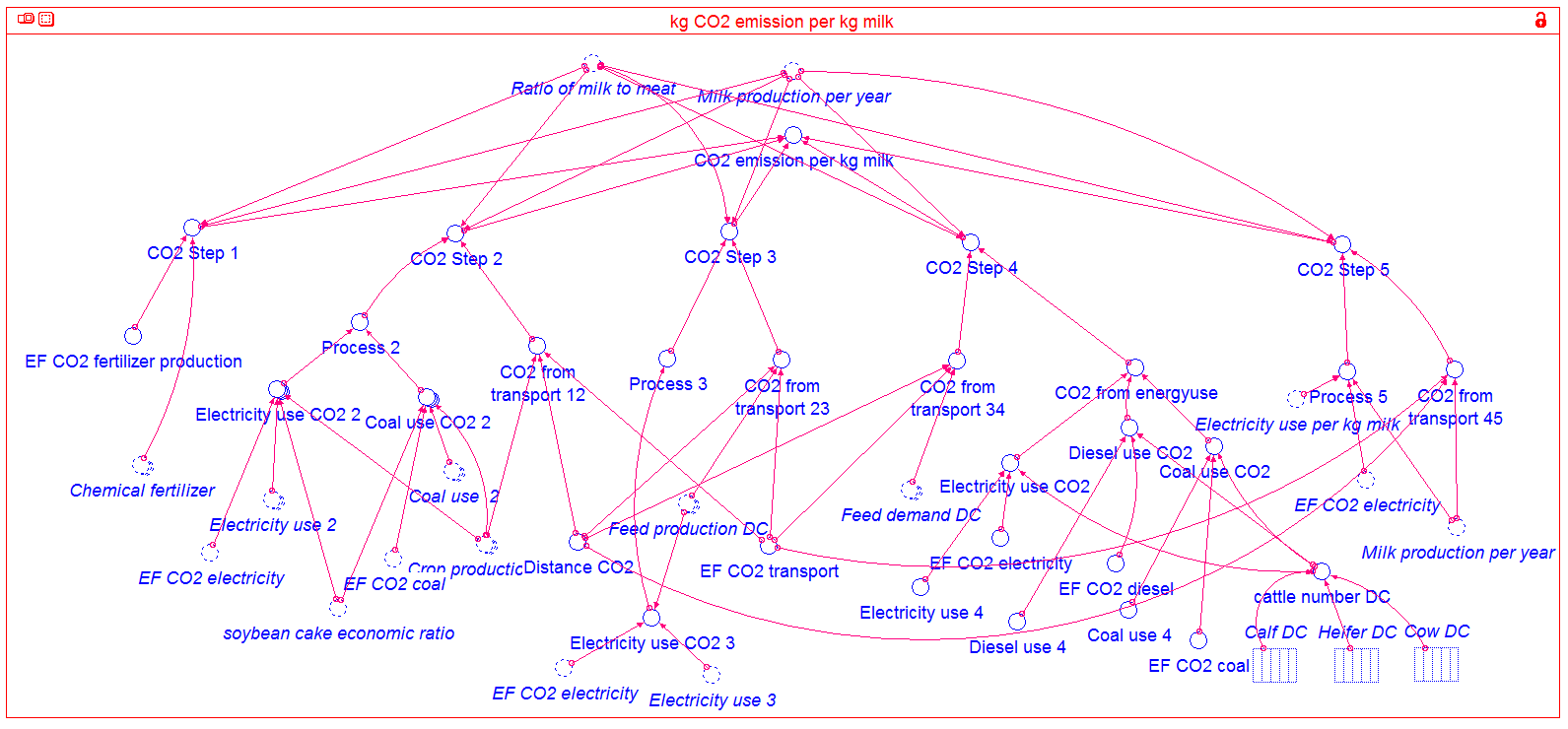


ca


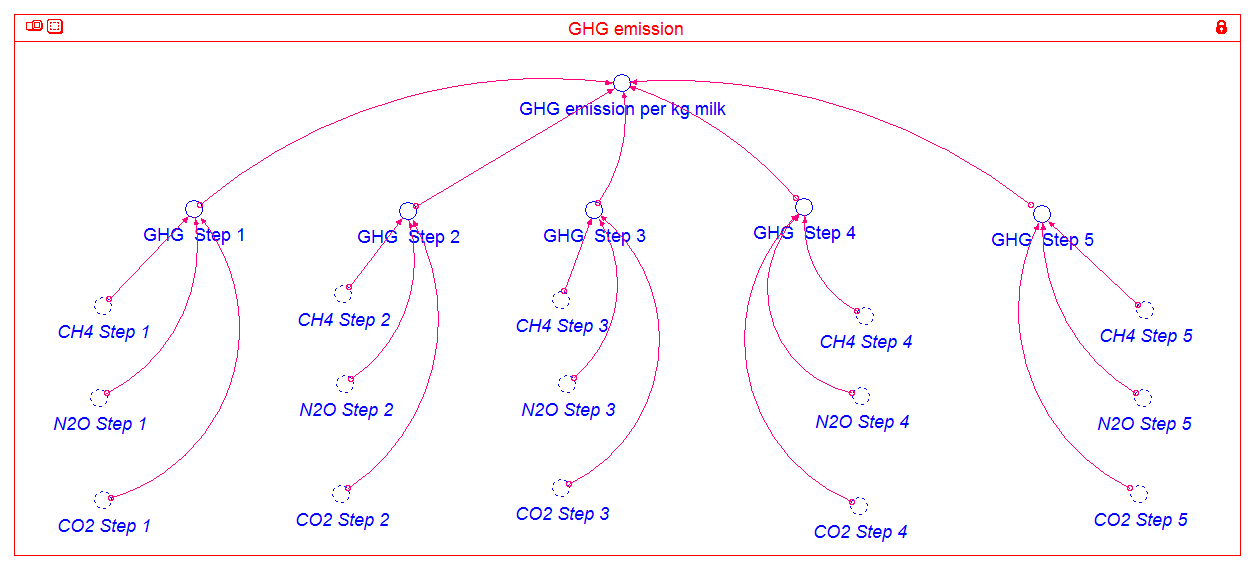


da

**Supplementary Figure 10 Diagram of the calculators for (a) CH_4_ (b) N_2_O (c) CO_2_ and (d) GHG emissions in STELLA graphic design (taking dairy cattle as an example).**


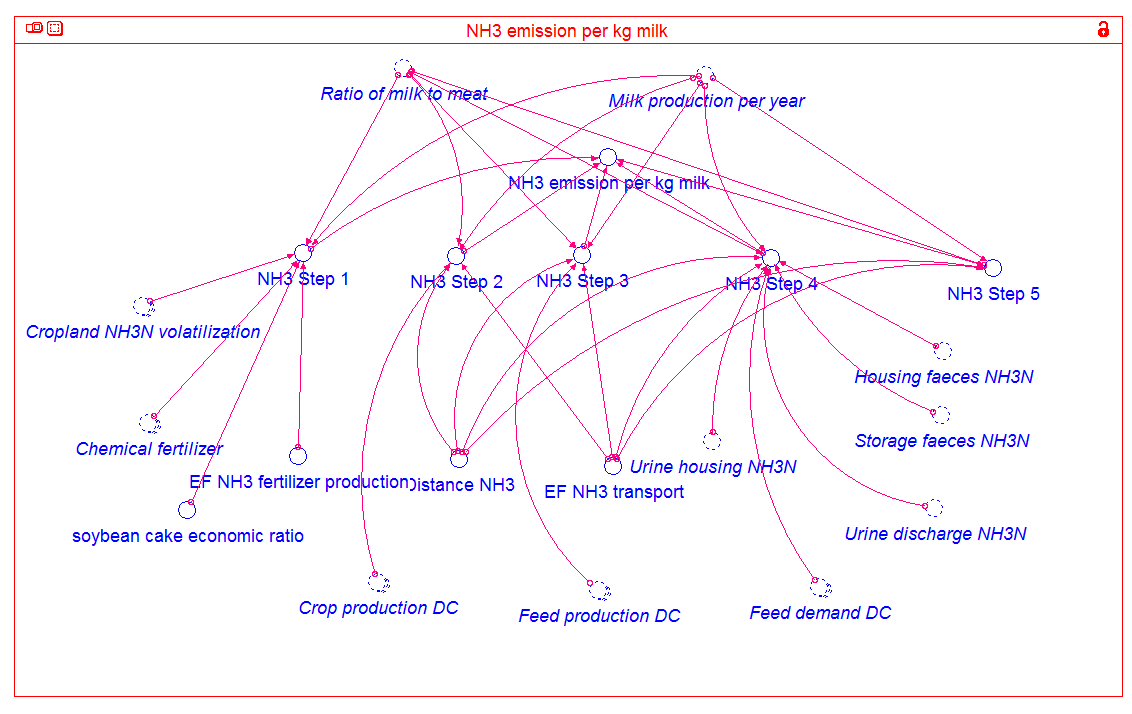


**Supplementary Figure 11 Diagram of NH_3_ emissions calculator in STELLA graphic design** **(taking beef cattle as an example).** Emissions of NH_3_ across the production chain come from N fertilizer production and application (here we considered urea as the chemical fertilizer used), transportation, ammonia emissions from livestock farming and manure management in barns, ammonia volatilization from organic fertilizer application, and the direct discharge of liquid manure.

**
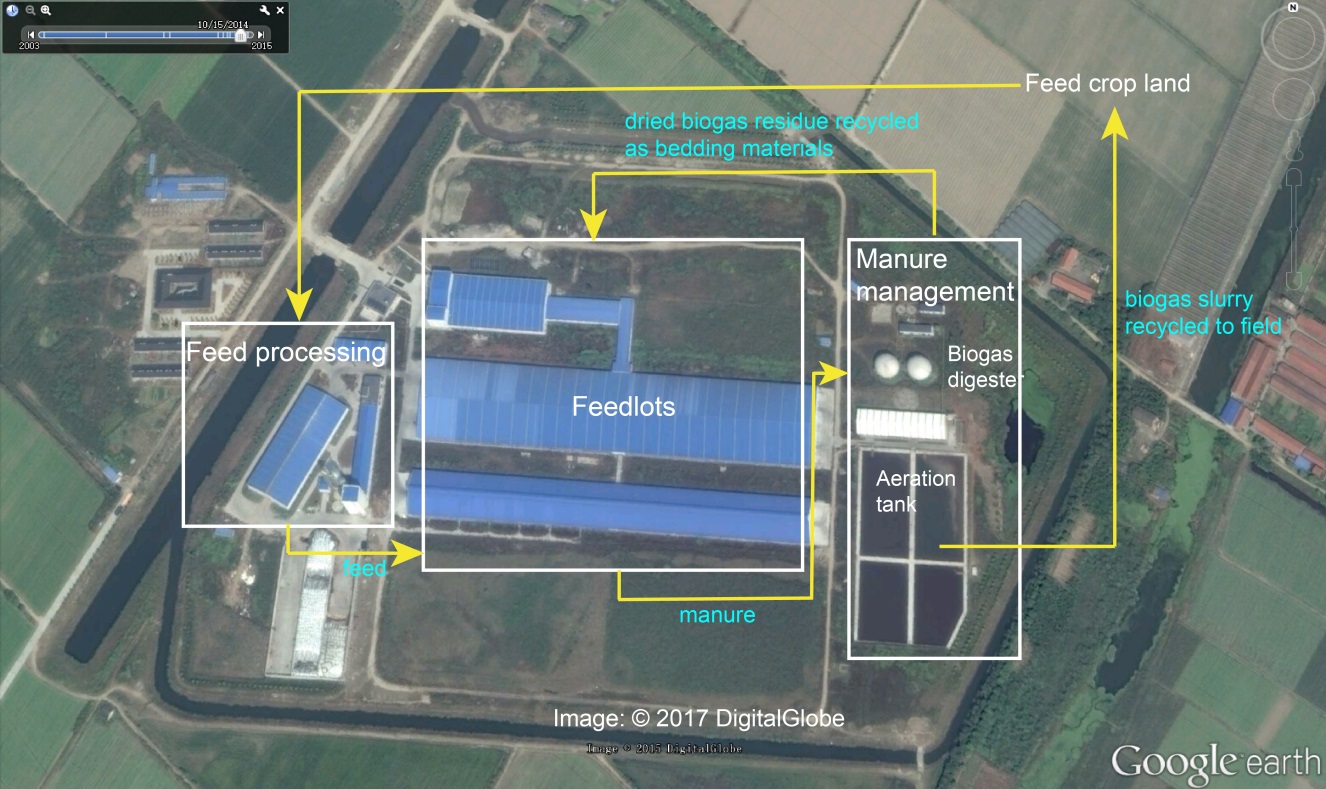
**

**Supplementary Figure 11 Yijing dairy farm a well-managed dairy farm in China located in Shaoxing City (30.1°N, 120.5°E) Zhejiang Province, Southeast China.** Arrows show material flows between different components of the farm system. The image is from Google Earth (version 7.1.5.1557).

**Supplementary Tables**

**Supplementary Table 1 Variation in production, feed, and manure managements for 166 dairy farms surveyed in this study.**

| **Characteristics** | **Grazing system** | **Mixed system** | **Industrial system** |
| --- | --- | --- | --- |
| **Production** |  |  |  |
| Size (head) | 5-100 | 35-180 | 200-17000 (50% of the feedlots have over 1000 head) |
| Milk yield (t head^-1^ yr^-1^) | 4 | 3-5.5 | 4.8-10 |
| Dairy herd improvement | 0 | 0 | 39% |
| Total mixed rations | 0 | 0 | 66% |
| **Feed** |  |  |  |
| Import (percent of farms) | 0 | 10% | 58% |
| Feed importing source | 0 | Alfalfa (USA) | Alfalfa (USA, Spain), Oatgrass (Australia) |
| **Manure management** (percent of farms) |  |  |  |
| Manure collection |  |  |  |
| Solid-liquid separation | 0 | 25% | 41% |
| Manure treatment |  |  |  |
| Biogas fermentation (slurry) | 0 | 5% | 40% |
| Composting (solid manure) | 0 | 60% | 62% |
| Sedimentation tank (liquid manure) | 0 | 5% | 33% |
| Manure application |  |  |  |
| Solid manure application | 100% | 100% | 100% |
| Liquid manure application | 100% | 5% | 8% |

**Supplementary Table 2 Variation in production, feed, and manure managements for 82 beef farms surveyed in this study.**

| **Characteristics** | **Grazing system** | **Mixed system** | **Industrial system** |
| --- | --- | --- | --- |
| **Production** |  |  |  |
| Size (head) | 15-90 | 40-190 | 200-6000 |
| Live weight before slaughter (kg head^-1^) | 230-300 | 400-450 | 500-750 |
| **Feed** |  |  |  |
| Import (percent of farms) | 0 | 0 | 0 |
| **Manure management** (percent of farms) |  |  |  |
| Manure collection |  |  |  |
| Solid-liquid separation | 0 | 0 | 0 |
| Manure treatment |  |  |  |
| Biogas fermentation (slurry) | 0 | 12% | 40% |
| Composting (solid manure) | 0 | 76% | 85% |
| Sedimentation tank (liquid manure) | 0 | 0 | 14% |
| Manure application |  |  |  |
| Solid manure application | 100% | 100% | 100% |
| Liquid manure application | 100% | 3% | 6% |

**Supplementary Table 3 Variation in production, feed, and manure management characteristics for 60 sheep farms surveyed in this study.**

| **Characteristics** | **Grazing system** | **Mixed system** | | **Industrial system** |
| --- | --- | --- | --- | --- |
| **Production** |  |  |  | |
| Size (head) | 26-88 | 96-135 | 460-1000 | |
| Live weight before slaughter (kg head^-1^) | 20-40 | 25-50 | 50-70 | |
| **Feed** |  |  |  | |
| Import (percent of farms) | 0 | 0 | 0 | |
| **Manure management** (percent of farms) |  |  |  | |
| Manure collection |  |  |  | |
| Slatted floor | 0 | 45% | 80% | |
| Manure treatment |  |  |  | |
| Biogas fermentation (slurry) | 0 | 0 | 10% | |
| Composting (solid manure) | 0 | 55% | 55% | |
| Sedimentation tank (liquid manure) | 0 | 0 | 0 | |
| Manure application |  |  |  | |
| Solid manure application | 100% | 40% | 50% | |
| Liquid manure application | 100% | 0 | 10% | |

**Supplementary Table 4 Initial values of parameters used in the N flux calculator**

| **Parameter** | Maize | Soybean | Alfalfa | Green maize | |  |
| --- | --- | --- | --- | --- | --- | --- |
| **Step 1-Feed crop planting**  Chemical fertilizer (kg N ha^-1^ yr^-1^) | Supplementary Table 9 | Supplementary Table 9 | Supplementary Table 9 | | Supplementary Table 9 | |
| Biological fixation (kg N ha^-1^ yr^-1^) | 0.00 | 115.00 | 345.00 | 0.00 | |  |
| Atmospheric deposition (kg N ha^-1^ yr^-1^) | 30.04 | 23.45 | 60.0 | 30.04 | |  |
| Irrigation water (kg N ha^-1^ yr^-1^) | 2.78 | 1.93 | 7.38 | 3.67 | |  |
| Purchased seeds (kg N ha^-1^ yr^-1^) | 2.78 | 1.93 | 7.38 | 3.67 | |  |
| Nitrogen use efficiency (%) | 12.39 | 45.98 | 18.89 | 9.10 | |  |
| **Step 2-Primary feed processing** | | | | | |  |
| Nitrogen loss ratio (%) | 1 | 1 | 1 | 1 | |  |
| **Step 3-Complete feed processing** | | | | | |  |
| Nitrogen loss ratio (%) | 1 | 1 | 1 | 1 | |  |
| **Step 4-Livestock raising** | | | | | |  |
| Nitrogen loss from feed mix (%) | 0.5 | 0.5 | 0.5 | 0.5 | |  |
| **Step 5-Livestock products processing** | Livestock products | | | | |  |
| *Dairy cattle* | Milk | | | | |  |
| Milk processing nitrogen loss ratio (%) | 2% | | | | |  |
| *Beef cattle* | Beef meat (live cattle to carcass) | | | | |  |
| Beef slaughter nitrogen loss ratio (%) | 55% | | | | |  |

**Supplementary Table 5 Initial values of parameters used in the GHG emissions calculator**

| **Parameter description** | **Unit** | **Values** | **Description of values** | **Data sources** |
| --- | --- | --- | --- | --- |
| **Step 1-Feed crop planting** |  |  |  |  |
| N_2_O emission from chemical fertiliser production | kg N_2_O kg^-1^ fertilizer N | 1.09× 10^-3^ | Data for urea | *10* |
| CO_2_ emission from chemical fertilizer production and transportation | kg CO_2_ kg-1 fertilizer N | 7.58 | Data for urea | *10* |
| CH_4_ emission from chemical fertilizer production and transportation | kg CH_4_ kg^-1^ fertilizer N | 26.45× 10^-3^ | Data for urea | *10* |
| Direct N_2_O emission from fertilizer application | kg N_2_O-N kg^-1^ applied N | 0.0105 | - | *11* |
| Indirect N_2_O emission from leaching or runoff of applied chemical fertiliser | kg N_2_O-N kg^-1^ N | 0.0075 | - | *11* |
| Indirect N_2_O emission factor due to the volatilization of NH_3_ and NO_x_ | kg N_2_O-N kg^-1^ N | 0.01 | - | *11* |
| Direct N_2_O emission during manure application | kg N_2_O-N kg^-1^ N | 0.01 | - | *11* |
| Indirect N_2_O emission from atmospheric nitrogen deposition, NH_3_-N and NO_x_-N of manure recycled to field | kg N_2_O-N kg^-1^ N | 0.002 | - | *11* |
| **Step 2-Feed primary processing** |  |  |  |  |
| Energy use in feed primary processing |  | Supplementary Table 6 |  |  |
| Emission factors of different energy |  | Supplementary Table 7 |  |  |
| CO_2_ emission factor for transportation | kg CO_2_ kg^-1^ km^-1^ | 0.179× 10^-3^ | By truck | *10* |
| **Step 3-Complete feed processing** |  |  |  |  |
| Energy use in completed feed processing |  | Supplementary Table 6 |  |  |
| Emission factors of different energy |  | Supplementary Table 7 |  |  |
| CO_2_ emission factor for transportation | kg CO_2_ kg^-1^ km^-1^ | 0.179× 10^-3^ | By truck | *10* |
| **Step 4-Livestock raising** |  |  |  |  |
| ***Dairy cattle*** |  |  |  |  |
| CH_4_ emission from enteric fermentation | kg CH_4_ head^-1^ yr^-1^ | (i) calf: 36.8; heifer: 52.0; adult cow: 71.7; (ii): calf: 37.3; heifer: 53.1; adult cow: 79.3 | (i) grazing system; (ii) industrial and mixed system | *12* |
| CH_4_ emission from manure management | kg CH_4_ head^-1^ yr^-1^ | Supplementary Table 10 |  |  |
| Direct N_2_O emission from liquid manure | kg N_2_O-N kg^-1^ N | 0.02 |  | *11* |
| Direct N_2_O emission from solid manure | kg N_2_O-N kg^-1^ N | 0.005 |  | *11* |
| Energy use on farm |  | Supplementary Table 6 |  |  |
| Emission factors of different energy |  | Supplementary Table 7 |  |  |
| ***Beef cattle*** |  |  |  |  |
| CH_4_ emission from enteric fermentation | kg CH_4_ head^-1^ yr^-1^ | (i): 85.3 in average; (ii): 67.9 in average | (i) grazing system; (ii) industrial and mixed system | *13* |
| CH_4_ emission from manure management | kg CH_4_ head^-1^ yr^-1^ | Supplementary Table 10 |  |  |
| Direct N_2_O emission from liquid manure | kg N_2_O-N kg^-1^ N | 0.02 |  | *11* |
| Direct N_2_O emission from solid manure | kg N_2_O-N kg^-1^ N | 0.005 |  | *11* |
| Energy use on farm |  | Supplementary Table 6 |  |  |
| Emission factors of different energy |  | Supplementary Table 7 |  |  |
| **Step 5-Livestock products processing** | |  |  |  |
| Energy use in processing |  | Supplementary Table 6 |  |  |
| Emission factor of different energy |  | Supplementary Table 7 |  |  |
| CO_2_ emission factor for transportation | kg CO_2_ kg^-1^ km^-1^ | 0.179× 10^-3^ | By truck | *10* |

**Supplementary Table 6 The energy use in different ruminant production systems.**

|  | **Grazing system** | **Mixed system** | **Industrial system** | **Unit** | **Source** |
| --- | --- | --- | --- | --- | --- |
| **Primary feed processing** | |  |  |  |  |
| *Electricity* |  |  |  |  |  |
| Maize | - | 39.60 | 39.60 | kW·h t^-1^ DM | *14* |
| Soybean | - | 25.00 | 25.00 | kW·h t^-1^ DM | *14* |
| Maize silage | - | 0.46 | 0.46 | kW·h t^-1^ DM | *15* |
| Alfalfa | - | 0.42 | 0.42 | kW·h t^-1^ DM | *15* |
| *Diesel* |  |  |  |  |  |
| Maize | - | 4.6 | 4.6 | kg t^-1^ DM | *14* |
| Soybean | - | 14.5 | 14.5 | kg t^-1^ DM | *14* |
| **Completed feed processing** | |  |  |  |  |
| *Electricity* | - | 5 | 5 | kW·h t^-1^ DM |  |
| **Livestock breeding** |  |  |  |  |  |
| *Electricity* |  |  |  |  |  |
| Dairy cattle | 118.68 | 370.98 | 482 | kW·h head^-1^ yr^-1^ | *14, 16* |
| Beef cattle | 16.5 | 33 | 66 | kW·h head^-1^ year^-1^ | *17, 18* |
| *Coal* |  |  |  |  |  |
| Dairy cattle | 68 | 87 | 68 | kg head^-1^ yr^-1^ | *16* |
| Beef cattle | 5 | 5 | 10 | kg head^-1^ yr^-1^ |  |
| *Diesel* |  |  |  |  |  |
| Dairy cattle | - | - | 32.7 | kg head^-1^ yr^-1^ |  |
| Beef cattle | - | - | - | kg head^-1^ yr^-1^ |  |
| **Livestock products processing** | |  |  |  |  |
| *Electricity* |  |  |  |  |  |
| Dairy cattle | 0 | 54 | 54 | kW·h t^-1^ milk |  |
| Beef cattle | 23.12 | 46.24 | 46.24 | kW·h head^-1^ | *17* |

**Supplementary Table 7 The emissions of energy use.**

|  | **Grazing system** | | **Mixed system** | | **Industrial system** | | **Unit** | | **Source** | |
| --- | --- | --- | --- | --- | --- | --- | --- | --- | --- | --- |
| **Electricity** |  | |  | |  | |  | |  | |
| CH_4_ emission factors for electricity generation | 0.0183×10^-3^ | | 0.01735×10^-3^ | | 0.01326×10^-3^ | | kg CH_4_ (kW·h)^-1^ | | *19* | |
| N2O emission factors for electricity generation | 0.01397×10^-3^ | | 0.01316× 10^-3^ | | 0.0796×10^-3^ | | kg N_2_O (kW·h)^-1^ | | *19* | |
| CO2 emission factors for electricity generation | 1.1967 | | 1.16918 | | 0.8508 | | kg CO_2_ (kW·h)^-1^ | | *19* | |
| **Coal** |  | |  | |  | |  | |  | |
| CH4 emission factors for coal mining | 9.32×10^-3^ | | 9.32×10^-3^ | | 9.32×10^-3^ | | kg kg^-1^ | | *20* | |
| CO2 emission factors for coal mining and combustion | 1.98 | | 1.98 | | 1.986 | | kg kg^-1^ | | *16* | |
| **Diesel** |  | |  | |  | |  | |  | |
| CH4 emission factors for diesel oil production | 0.215×10^-3^ | | 0.215×10^-3^ | | 0.215×10^-3^ | | kg kg^-1^ | | *20* | |
| CO2 emission factors for diesel oil production and combustion | | 2.76 | | 2.76 | | 3.38 | | kg kg^-1^ | | *20* |

**Supplementary Table 8 Initial values of parameters used in the NH_3_ emissions calculator**

| **Parameter description** | **Unit** | **Values** | **Description of values** | **Data sources** |
| --- | --- | --- | --- | --- |
| **Step 1-Feed crop planting** |  |  |  |  |
| NH_3_ emission factor for chemical fertilizer production | kg NH_3_ kg^-1^ fertilizer N | 0.01 | Data for urea that is the main N fertilizer used in China, and the N content of urea is ~46% | *21* |
| NH_3_ emission factor for chemical fertilizer application | kg NH_3_-N kg^-1^ fertilizer N | South of China: 0.172; north of China: 0.25 | - | *22, 23* |
| NH_3_ emission factor for manure application | kg NH_3_-N kg^-1^ manure N | South of China: 0.099; north of China: 0.25 | - | *22, 23* |
| NH_3_ emission factor for biological N fixation | kg NH_3_ kg^-1^ fixed N | 0.01 | - |  |
| **Step 2-Feed primary processing** |  |  |  |  |
| NH_3_ emission factor for transportation | g NH_3_ km^-1^ | 0.026 | Light-duty gasoline vehicles | *21* |
| The allocation factor of feed | - | Grain: 1.0; soybean-cake: 0.66 | - | *24* |
| **Step 3-Complete feed processing** |  |  |  |  |
| NH_3_ emission factor for transportation | g NH_3_ km^-1^ | 0.026 | Light-duty gasoline vehicles | *21* |
| **Step 4-Livestock raising** |  |  |  |  |
| ***Dairy cattle*** |  |  |  |  |
| Fraction of TAN from urine | - | 60% | - | *21* |
| Fraction of TAN from faeces | - | 60% | - | *21* |
| NH_3_ emission factor for urine in housing | kg NH_3_-N kg^-1^ TAN | (i) 0.158; (ii) 0.14; (iii) 0.15 | (i) grazing system; (ii) mixed system; (iii) industrial | *21* |
| NH_3_ emission factor for faeces in housing | kg NH_3_-N kg^-1^ TAN | (i) 0.158; (ii) 0.14; (iii) 0.15 | (i) grazing system; (ii) mixed system; (iii) industrial | *21* |
| NH_3_ emission factor for solid manure in storage/treatment | kg NH_3_-N kg^-1^ TAN | (i) 0.27 ; (ii) 0.27 ; (iii) 0.042 | (i) grazing system; (ii) mixed system; (iii) industrial | *11* |
| NH_3_ emission of direct discharged liquid manure | kg NH_3_-N kg^-1^ TAN | 0.3 | Liquid manure is not managed and fully exposed to the air | *11* |
| ***Beef cattle*** |  |  |  |  |
| Fraction of TAN from urine | - | 60% | - | *21* |
| Fraction of TAN from faeces | - | 60% | - | *21* |
| NH_3_ emission factor for urine in housing | kg NH_3_-N kg^-1^ TAN | (i) 0.093; (ii) 0.14; (iii) 0.14 | (i) grazing system; (ii) mixed system; (iii) industrial | *21* |
| NH_3_ emission factor for faeces in housing | kg NH_3_-N kg^-1^ TAN | (i) 0.093; (ii) 0.14; (iii) 0.14 | (i) grazing system; (ii) mixed system; (iii) industrial | *21* |
| NH_3_ emission factor for solid manure in storage/treatment | kg NH_3_-N kg^-1^ TAN | (i) 0.27 ; (ii) 0.27 ; (iii) 0.042 | (i) grazing system; (ii) mixed system; (iii) industrial | *21* |
| NH_3_ emission of direct discharged liquid manure | kg NH_3_-N kg^-1^ TAN | 0.55 | Liquid manure is not managed and fully exposed to the air | *21* |
| **Step 5-Livestock products processing** | |  |  |  |
| The allocation factor of raw milk | - | 0.92 | - | *This study* |
| NH_3_ emission factor for transportation | g NH_3_ km^-1^ | 0.026 | Light-duty gasoline vehicles | *21* |

**Supplementary Table 9 Rates of fertilizer N application and yield of main feed types in the provinces of mainland China**

| Province | Nitrogen application (kg N ha^-1^ yr^-1^) | | | |  | Yield (kg ha^-1^ yr^-1^) | | | |
| --- | --- | --- | --- | --- | --- | --- | --- | --- | --- |
|  | Maize^a^ | Soybean^a^ | Alfalfa^b^ | Green maize^c^ |  | Maize^a^ | Soybean^a^ | Alfalfa^d^ | Green maize^c^ |
| Inner Mongolia | 229.4 | 46.8 | 92.5 | 706.8 |  | 7979.7 | 1955.0 | 13689.2 | 30300.0 |
| Tibet | 266.1 | 46.8 | 99.8 | 164.1 |  | 8884.2 | 1955.0 | 11653.1 | 6750.0 |
| Qinghai | 266.1 | 46.8 | 103.5 | 358.4 |  | 8884.2 | 1955.0 | 6720.0 | 14745.0 |
| Xinjiang | 302.9 | 46.8 | 103.5 | 462.9 |  | 9788.7 | 1955.0 | 14550.0 | 18435.0 |
| Hubei | 258.0 | 21.3 | 123.0 | 619.2 |  | 6485.0 | 1881.4 | 9922.6 | 19176.4 |
| Hunan | 230.9 | 21.3 | 123.0 | 639.6 |  | 6188.9 | 1881.4 | 9922.6 | 21120.0 |
| Guangxi | 252.2 | 21.3 | 123.0 | 753.8 |  | 5253.3 | 1881.4 | 9922.6 | 19350.0 |
| Hainan | 230.9 | 21.3 | 123.0 | 580.8 |  | 6188.9 | 1881.4 | 9922.6 | 19176.4 |
| Chongqing | 215.8 | 14.1 | 60.0 | 397.9 |  | 6226.7 | 1745.4 | 8394.2 | 14145.0 |
| Guizhou | 191.2 | 21.3 | 123.0 | 863.0 |  | 3448.2 | 1881.4 | 9922.6 | 19176.4 |
| Yunnan | 289.7 | 21.3 | 67.5 | 841.5 |  | 5356.8 | 1881.4 | 2623.3 | 19176.4 |
| Anhui | 215.7 | 28.6 | 123.0 | 789.3 |  | 6551.0 | 2017.4 | 9922.6 | 29535.0 |
| Fujian | 230.9 | 21.3 | 123.0 | 231.7 |  | 6188.9 | 1881.4 | 9922.6 | 7650.0 |
| Jiangxi | 230.9 | 21.3 | 123.0 | 636.0 |  | 6188.9 | 1881.4 | 9922.6 | 21000.0 |
| Sichuan | 194.1 | 21.3 | 241.5 | 527.5 |  | 6401.9 | 1881.4 | 18750.2 | 21435.0 |
| Liaoning | 210.2 | 63.2 | 75.0 | 456.1 |  | 7012.8 | 2736.9 | 11850.0 | 18750.0 |
| Jilin | 198.6 | 75.4 | 95.5 | 702.1 |  | 7979.7 | 2547.5 | 4266.9 | 34770.0 |
| Heilongjiang | 141.3 | 62.1 | 27.0 | 308.5 |  | 6904.1 | 2245.4 | 11538.2 | 18570.0 |
| Hebei | 156.1 | 39.2 | 250.0 | 474.3 |  | 7199.3 | 2283.3 | 10235.8 | 26955.0 |
| Shandong | 213.0 | 63.0 | 225.4 | 1012.7 |  | 7167.2 | 2495.1 | 21333.3 | 41985.0 |
| Gansu | 298.5 | 63.0 | 334.5 | 513.4 |  | 9832.1 | 2323.6 | 12871.2 | 20835.0 |
| Ningxia | 316.0 | 63.0 | 54.0 | 471.6 |  | 8149.8 | 2323.6 | 10149.2 | 14985.0 |
| Henan | 170.4 | 16.1 | 276.0 | 502.3 |  | 6638.4 | 2042.7 | 15097.3 | 24120.0 |
| Shaanxi | 275.4 | 95.0 | 159.5 | 828.6 |  | 6267.9 | 1876.4 | 15472.3 | 23236.7 |
| Shanxi | 180.0 | 90.5 | 11.3 | 135.7 |  | 8784.2 | 2361.8 | 3607.0 | 8160.0 |
| Beijing | 263.0 | 54.7 | 50.0 | 988.0 |  | 8500.0 | 2194.8 | 13406.7 | 39345.0 |
| Tianjin | 263.0 | 54.7 | 70.5 | 410.2 |  | 8500.0 | 2194.8 | 11813.0 | 16335.0 |
| Shanghai | 247.7 | 54.7 | 93.9 | 875.5 |  | 5787.8 | 2194.8 | 12725.6 | 25211.3 |
| Jiangsu | 247.7 | 54.7 | 120.0 | 422.5 |  | 5787.8 | 2194.8 | 20200.0 | 12165.0 |
| Zhejiang | 247.7 | 54.7 | 135.0 | 875.5 |  | 5787.8 | 2194.8 | 5482.5 | 25211.3 |
| Guangdong | 247.7 | 54.7 | 93.9 | 1146.0 |  | 5787.8 | 2194.8 | 12725.6 | 33000.0 |

a: N application rates and yields of maize and soybean are from ref 63;

b: data for alfalfa are from refs 64-70.

c: data on green maize yields are from ref 71, and the N application rates are calculated with the assumption that green maize and grain maize have the same N use efficiency in the same region.

**Supplementary Table 10 The CH_4_ emission factors of manure management for beef and dairy cattle in provices of China.**

| Province | Beef cattle | Dairy cattle |
| --- | --- | --- |
|  | kg CH_4_ head^-1^ yr^-1^ | kg CH_4_ head^-1^ yr^-1^ |
| Beijing | 6.620 | 8.508 |
| Tianjin | 7.161 | 10.127 |
| Hebei | 2.654 | 5.889 |
| Shanxi | 2.246 | 2.776 |
| Inner Mongolia | 1.245 | 5.755 |
| Liaoning | 0.611 | 4.813 |
| Jilin | 0.581 | 1.456 |
| Heilongjiang | 0.459 | 2.073 |
| Shanghai | - | 19.117 |
| Jiangsu | 1.785 | 17.680 |
| Zhejiang | 1.414 | 19.421 |
| Anhui | 1.375 | 12.740 |
| Fujian | 1.860 | 15.278 |
| Jiangxi | 2.506 | 17.693 |
| Shandong | 2.877 | 7.947 |
| Henan | 1.149 | 9.140 |
| Hubei | 1.795 | 12.860 |
| Hunan | 1.823 | 15.645 |
| Guangdong | 1.890 | 16.669 |
| Guangxi | 1.678 | 22.107 |
| Hainan | 2.867 | 14.348 |
| Chongqing | - | - |
| Sichuan | 1.387 | 17.018 |
| Guizhou | 2.235 | 10.211 |
| Yunnan | 1.359 | 9.670 |
| Tibet | 1.433 | 8.985 |
| Shaanxi | 1.443 | 14.319 |
| Gansu | 1.061 | 9.964 |
| Qinghai | 1.486 | 8.419 |
| Ningxia | 0.815 | 2.520 |
| Xinjiang | 1.357 | 11.797 |

Data source: ref. 12.

**Supplementary Table 11 The demand projections for ruminant meat and dairy products into the future. Unit: kg capita^-1^ yr^-1^**.

| Item | Year | Business-as-usual | Tilman approach^4,5^ | Alexandratos approach^6^ | Havlík approach | | | Average |
| --- | --- | --- | --- | --- | --- | --- | --- | --- |
|  |  |  |  |  | Ward et al., 2012^7^ | Meyer et al., 2012^8^ | Hawksworth, 2006^9^ |  |
| Milk | 2020 | 44.4 | 145.6 | 56.8 | 36.8 | 35.8 | 33.8 | 58.9 |
|  | 2030 | 54.8 | 157.9 | 60.9 | 43.1 | 40.7 | 36.7 | 65.7 |
|  | 2040 | 65.2 | 167.9 | 64.1 | 45.4 | 42.3 | 37.8 | 70.5 |
|  | 2050 | 75.6 | 186.7 | 66.7 | 49.8 | 46.0 | 41.9 | 77.8 |
| Ruminant meat | 2020 | 9.4 | 18.2 | 12.9 | 10.6 | 11.1 | 9.8 | 12.0 |
|  | 2030 | 11.0 | 19.8 | 13.9 | 13.4 | 12.7 | 11.5 | 13.7 |
|  | 2040 | 12.7 | 20.3 | 14.7 | 16.9 | 15.8 | 14.1 | 15.7 |
|  | 2050 | 14.4 | 18.94 | 15.3 | 21.1 | 19.5 | 17.7 | 17.8 |

Note: Following Havlík^82^, we used three GDP forecasts based on Ward et al., 2012, Meyer et al., 2012, and Hawksworth, 2006, corresponding to reference *7-9*.

**Supplementary Table 12 Damage costs to ecosystem and human health from GHG and NH_3_ emissions in China and the exporting nations.**

| Emission in China | Quantity | Ecosystem health | Human health | Emission in exporting nations | Quantity | Ecosystem health | Human health |
| --- | --- | --- | --- | --- | --- | --- | --- |
|  | Mt | US$ | US$ |  | Mt | US$ | US$ |
| GHG | 269.75 | 3.22E+10 | 6.20E+08 | GHG | 12 | 1.43E+09 | 2.76E+07 |
| NH_3_ | 2.34 | 3.44E+08 | 2.17E+10 | NH_3_ | 0.04 | 5.89E+06 | 3.71E+08 |

**Supplementary Table 13 Conversion coefficients to midpoint and endpoint impacts of increasing GHG and NH_3_ emissions.** Damage to human health (HH) is expressed as disability-adjusted loss of life years (DALY) and the damage to ecosystem health (EH) by biodiversity-adjusted hectare years (BAHY)

| **Emission** | **Midpoint impact** | **Coefficients** | **Unit** | **Ref** | **Endpoint impact** | **Coefficients** | **Unit** | **Ref** |
| --- | --- | --- | --- | --- | --- | --- | --- | --- |
| CO_2_ | Climate change | 1 | kg CO_2_-eq kg^-1^ | *25* | HH | 2.10E-08 | DALY (kg CO_2_-eq)^-1^ | *26* |
|  | Climate change | 1 | kg CO_2_-eq kg^-1^ | *25* | EH | 5.77E-05 | BAHY (kg CO_2_-eq)^-1^ | *27* |
| CH_4_ | Climate change | 28 | kg CO_2_-eq kg^-1^ | *25* | HH | 2.10E-08 | DALY (kg CO_2_-eq)^-1^ | *26* |
|  | Climate change | 28 | kg CO_2_-eq kg^-1^ | *25* | EH | 5.77E-05 | BAHY (kg CO_2_-eq)^-1^ | *27* |
| N_2_O | Climate change | 265 | kg CO_2_-eq kg^-1^ | *25* | HH | 2.10E-08 | DALY (kg CO_2_-eq)^-1^ | *26* |
|  | Climate change | 265 | kg CO_2_-eq kg^-1^ | *25* | EH | 5.77E-05 | BAHY (kg CO_2_-eq)^-1^ | *27* |
| NH_3_ | Particulate matter formation | 0.121 | kg PM_2.5_-eq kg^-1^ | *26* | HH | 7.00E-04 | DALY (kg PM_2.5_-eq)^-1^ | *26* |
|  | Terrestrial acidification | 6.4 | m^2^ UES kg^-1^ | *26* | EH | 5.50E-06 | BAHY (m^2^ UES)^-1^ | *26* |
|  | Marine eutrophication | 0.112 | kg N-eq kg^-1^ | *26* | EH | 3.20E-04 | BAHY (kg N-eq)^-1^ | *26* |

**Supplementary Table 14 GHG and NH_3_ emissions in the whole livestock production chain of exporting nations.**

| **Item** | **Nations** | **GHG**  **(kg CO_2_-eq kg product^-1^)** | **Reference** | **NH_3_**  **(kg NH_3_ t product^-1^)** | **Reference** |
| --- | --- | --- | --- | --- | --- |
|  |  |  |  |  |  |
| Maize | USA | 0.23 | *28* | 1.80 | *28* |
| Soybean | USA | 0.62 | *29* | 0.40 | *30* |
|  | Brazil | 0.62 | *29* | 0.40 | *30* |
|  | Argentina | 0.62 | *29* | 0.00 | *31* |
| Alfalfa | USA | 0.62 | *29* | 0.40 | *30* |
| Ruminant meat | New Zealand | 12.14 | *29* | 83.50 | *32* |
|  |  |  |  |  |  |
|  | Australia | 12.00 | *33* | 83.50 | *32* |
|  | Uruguay | 14.3 | *34* | 115.00 | *32* |
| Dairy | USA | 1.13 | *35* | 8.60 | *38* |
|  | Australia | 1.11 | *36* | 4.72 | *39* |
|  | New Zealand | 1.00 | *37* | 3.28 | *39* |

**Supplementary Table 15 Percentage of imports of ruminant products and livestock feed from major trade partners of China in 2012, and the average distance travelled.**

| **Item** | **Percentage of import** |  | **Destination port** |  | **Exportation port** | **Distance (km)** |
| --- | --- | --- | --- | --- | --- | --- |
|  |  |  |  |  |  |  |
| Maize | 100 |  | Shanghai (CN) |  | Los Angeles (US) | 10556 |
| Soybean | 44 |  | Shanghai (CN) |  | Los Angeles (US) | 10556 |
|  | 41 |  | Shanghai (CN) |  | Rio De Janeiro (BR) | 20196 |
|  | 15 |  | Shanghai (CN) |  | Buenos Aires (AR) | 20388 |
| Alfalfa | 100 |  | Shanghai (CN) |  | Los Angeles (US) | 10556 |
| Meat* | 42 |  | Shanghai (CN) |  | Auckland (NZ) | 9495 |
|  | 42 |  | Shanghai (CN) |  | Sydney (AU) | 8436 |
|  | 16 |  | Shanghai (CN) |  | Montevideo (UY) | 20236 |
| Dairy | 6 |  | Shanghai (CN) |  | Los Angeles (US) | 10556 |
|  | 8 |  | Shanghai (CN) |  | Sydney (AU) | 8436 |
|  | 86 |  | Shanghai (CN) |  | Auckland (NZ) | 9495 |

CN: China, US: United States, BR: Brazil, AR: Australia, NZ: New Zealand, UY: Uruguay, DE: Germany. We assume that all goods imported to China arrive at Shanghai port. *Ruminant meat

**Supplementary Table 16 Scenario settings and descriptions for each of the six scenarios described in Figure 4.** Note the addition of the two endpoint scenarios S5 (Total global supply) and S6 (Total domestic supply) in comparison to Figure 4.

|  | # | Description | Name | Green technology | Green-source trade |
| --- | --- | --- | --- | --- | --- |
| Baseline scenario | S0 | 1. Slow increase in China’s technology; 2. Small scale increase in China’s ruminant production in 2050;   (3) The ruminant product gap would be imported under current trading mode. | Business as usual | - | - |
| Green-source trade scenario | S1 | 1. Slow increase in China’s technology; 2. Small scale increase in China’s ruminant production in 2050;   (3) The **ruminant product** gap would be imported from nations with **low emission intensities**. | Globalized ruminant expansion | - | Ruminant products gap |
|  | S2 | 1. Slow increase in China’s technology; 2. **Large** scale increase in China’s ruminant production in 2050;   (3) The **feed** would be imported from nations with **low emission intensities**. | Globalized feed expansion | - | Feed |
| Technology scenario | S3 | (1) **Fast** increase in China’s technology;  (2) **Large** scale increase in China’s ruminant production in 2050;  (3) The ruminant product gap would be imported under current trading mode. | Localized technology improvement | Local ruminant production | - |
| Green-source trade & technology scenario | S4 | (1) **Fast** increase in China’s technology;  (2) **Large** scale increase in China’s ruminant production in 2050;  (3) The **ruminant product** gap would be imported from nations with **low emission intensities**. | Globalized sustainable intensification | Local ruminant production | Ruminant products gap |
| Endpoint scenarios | S5 | (1) All ruminant product demand met by imports from nations with **low emissions intensities**. | Total global supply | - | Ruminant products |
|  | S6 | (1) **Fast** increase in China’s technology;  (2) All ruminant product demand met by China’s domestic production. | Total domestic supply | Local ruminant production | - |

Note: Green-source for feed refers to import soybean, alfalfa, and maize that are demanded by local ruminant rearing from low emission nations. Green technology refers to China’s domestic GHG and NH_3_ emissions intensities decreased to the global-best practice, especially for industrial systems for they own the standard equipment and relatively higher potential to achieve technology improvement.

**Supplementary Table 17 Current approaches to manure management and application in China and the advanced technologies that could be adopted.**

| **Step** | **Current situation in China** | **Emission mitigation strategies** | | | | | |
| --- | --- | --- | --- | --- | --- | --- | --- |
|  |  | **Strategy** | **GHG abatement efficiency** | **Ref** | **Strategy** | **NH_3_ abatement efficiency** | **Ref** |
| Feed crop planting | (1) Only solid manure is used, very few of the farms use liquid manure.  (2) There is no upper limit on the amount of solid manure applied per unit area of cultivated land. | Timing of application | > 30% for CH_4_ | *58* | Injection–open slot | 60% | *59* |
|  |  | Soil nutrient balance | > 30% for N_2_O | *58* | Injection–closed slot | 80% | *59* |
|  |  | Nitrification inhibitor applied to manure | > 30% for N_2_O | *58* | Trailing hose | 20% | *60* |
|  |  |  |  |  | Trailing shoe | 60% | *60* |
|  |  |  |  |  | Cultivator | 80% | *60* |
|  |  |  |  |  | Urease inhibitor applied with or before urine | > 30% | *58* |
| Livestock raising | (1) The solid and liquid manure in the feedlot are not separated. In north China, the faeces from manual cleaning are directly stacked outdoors without any treatment. Most of the liquid manure is discharged into nearby rivers.  (2) A few of the farms are equipped with anaerobic digestion, but most of them have been abandoned for high operating cost. | Slurry acidification | 87% for CH_4_ | *61* | Slurry acidification | 65% (housing), 83% (outdoor storage) | *61* |
|  |  | Replacing grass silage with maize silage | reduce enteric CH_4_ emission | *61* | Concrete cover | 90% | *60* |
|  |  | Composting | 71% for CH_4_ and 49% for N_2_O | *61* | Flexible floating covers plastic sheet | 60% | *59* |
|  |  | Solids separation | > 30% for CH_4_ | *58* | Natural crust formation | 35%-50% | *59* |
|  |  | Aeration | > 30% for CH_4_ | *58* | Granulates | 85% | *60* |
|  |  | Sealed storage with flare | > 30% for CH_4_ and N_2_O | *58* | Thermal Drying with air scrubber | 85% | *62* |
|  |  | Aeration during liquid | 10%-30% for CH_4_ | *58* |  |  |  |

**Supplementary Table 18 Some subsidy policies for beef and sheep in China.**

| **Name** | **Policy and measures** |
| --- | --- |
| Yellow box policies | Subsidies for beef & sheep breeds |
|  | Funding support for standardized scale farms of beef & sheep |
|  | Compulsory immunization subsidy policy for major animal diseases |
| Green box policies | Development of quality and safety management traceability system |
|  | Livestock husbandry technical training, promotion and demonstration of science and technology |
|  | Grassland ecological conservation subsidy enthusiasm mechanism |
|  | Subsidy policy for agriculture insurance premium |

**Supplementary Table 19 Some subsidy policies for dairy cattle in China.**

| **Name** | **Policy and measures** |
| --- | --- |
| Yellow box policies | Subsidies for dairy cattle breeds |
|  | Implementation of dairy machinery subsidies |
| Green box policies | Subsidies for dairy cattle insurance |
|  | Funding support for dairy cattle production measurement traits |
|  | Funding support for quality and safety supervision project of raw milk |
|  | Funding support for dairy cattle standardized scale breeding construction |
|  | Implementation of high-yield and high-quality alfalfa demonstration project |

**Supplementary Table 20 The available export quantities from green-source trading nations.**

|  |  | **2007** | **2012** | **2050** |
| --- | --- | --- | --- | --- |
| Ruminant meat | Available for export (t) | 6262852 | 7081125 | 5993672 |
|  | Demand for imports in China (t) | 52050 | 201105 | 8302591 |
|  | Import demand / export availability (%) | 0.83 | 2.84 | 138.52 |
|  | Available for export (t) | 77438333 | 103230190 | 76165808 |
| Dairy products | Demand for imports in China (t) | 2161049 | 7864930 | 56036211 |
|  | Import demand / export availability (%) | 2.79 | 7.62 | 73.57 |

Note: The available amount for exporting is for green-source trade nation/regions, including Australia, New Zealand, EU, USA.

**Supplementary Table 21 Some free trade agreements involving China.** These free trade agreements stimulated a large quantity of ruminant products to be imported into China. Note that in July 2017, China will lift the import ban on US beef which was set in 2003 due to mad cow disease.

| **Free trade agreement** | **Implementation date** | **Member countries** | **Goods exporting to China** |
| --- | --- | --- | --- |
| First Agreement on Trade Negotiations among Developing Member Countries of the Economic and Social Commission for Asia and the Pacific | September 1, 2006 | China, Bangladesh, India, Laos, South Korea, Sri Lanka and Mongolia | Agricultural products, fuel, chemical products, etc. |
| Free Trade Agreement between China and Chile | October 1, 2006 | China, Chile | Beef and mutton, dairy products, fruits, seafood, leather, fiber products, etc. |
| Free Trade Agreement between China and New Zealand | October 1, 2008 | China, New Zealand | Dairy products, wool, meat, edible offal, fish, vegetables, fruits and nuts, grains etc. |
| Free Trade Agreement between China and Singapore | January 1, 2009 | China, Singapore | Dairy products, chicken, mutton, fish, fruit, wool, etc. |
| Free Trade Agreement between China and Pakistan | November 11, 2015 | China, Pakistan | Vegetables, fruit, leather, aquatic products, etc. |
| China and ASEAN Free Trade Area (CAFTA) | January 1, 2010 | China, Cambodia, Brunei, India, Laos, Malaysia, Myanmar, Indonesia, Philippines, Singapore, Thailand and Vietnam | Live animal, meat and edible meat offal, fish, dairy and other animal products, vegetables, fruit and nuts, etc. |
| Free Trade Agreement between China and Switzerland | July 1, 2014 | China, Switzerland | Agricultural products, textiles, clothing, shoes and hats, etc. |
| Free Trade Agreement between China and Iceland | July 1, 2014 | China, Iceland | Marine products and related processed products |
| Free Trade Agreement between China and Australia | December 20, 2015 | China, Australia | Agricultural products |
| Free Trade Agreement between China and South Korea | December 20, 2015 | China, South Korea | Meat, seafood, dairy products, beans, corn, vegetables, grains, fruits, etc. |

**Supplementary Table 22 Some policies related to land use of China in the past two decades.**

| **Policy** | **Released Year** | **Aims/Objectives** | **Source** |
| --- | --- | --- | --- |
| Natural Forest Conservation Program | 1998 | Halt logging/ deforestation and protect natural forests for ecological/carbon benefits via mountain closure, aerial seeding and artificial planting. | Ref. 77 |
| Grain for Green Program | 1999 | Prevent soil erosion, mitigate flooding, store carbon, and improve livelihoods by increasing forest and grassland cover on cropped hillslopes and converting cropland, barren hills and wasteland to forest. | Ref. 77 |
| Forest Ecosystem Compensation Fund | 2000 | Conserve natural forests and protect species and ecosystems via restoration, protection, and management of forests that have important ecological, biodiversity conservation, and sustainable economic and social value | Ref. 77 |
| Opinions of the state council on further promoting western development. | 2008 | Returning farmland to forest and pasture, natural forest protection, and returning cultivated grassland to grassland etc. | http://www.gov.cn/zhengce/content/2008-03/28/content_2060.htm |
| Grassland Ecological Protection Program | 2010 | Mitigate grassland degradation by grazing prohibition and enhancing grassland vegetation coverage or biomass. | Ref. 77 |
| Comprehensive deepening of rural reform to accelerate the promotion of agricultural modernization. | 2014 | Since 2014, returning farmland to forest and grassland has been carried out in steep slope farmland, severely desertified farmland and important water source areas. | http://www.gov.cn/zhengce/content/2014-03/10/content_8705.htm |
| National agricultural modernization program (2016 - 2020) | 2016 | Reduce maize cultivation area and promote grain to feed program in northeastern cold areas, northern agro-pastoral ecotone, northwestern dry aeolian sand region, the Taihang Mountain area and the southwest rocky desertification area. Restore and increase soybean area and expand the range of grain soybean rotation. | http://www.gov.cn/zhengce/content/2016-10/20/content_5122217.htm |
| "13th Five-Year" plan for national grassland protection, construction and utilization | 2016 | Propose the target of grassland protection to 2020. Effectively curb grassland degradation, improve grassland productivity. | http://www.moa.gov.cn/nybgb/2017/dyiq/201712/t20171227_6129885.htm |
| The National Program for the Planning of Land (2016-2030) | 2017 | The indices of forest coverage, grassland vegetation coverage and cultivated land holding amount in 2030 are listed. | http://www.gov.cn/zhengce/content/2017-02/04/content_5165309.htm. |
| Opinions of the State Council on implementing the division of work of priority departments of report on the work of the government. | 2017 | "Grain to forage" area has been expanded to more than 10 million mu, more than 12 million mu has been completed for returning farmland to forest and grass, to strengthen desertification and rock desertification control. | http://www.gov.cn/zhengce/content/2017-03/28/content_5181530.htm. |
| Major models of crop straw used for agriculture | 2017 | Promote the comprehensive utilization of crop straw, such as silage for feeding livestock. | http://www.moa.gov.cn/nybgb/2017/dwq/201712/t20171230_6133475.htm |
| Implementation of the program of changing grain to forage policy | 2017 | Push the transformation of planting structure to grain-cash-forage crops pattern, and build up a combination of grain and grass. The new structure of agriculture and animal husbandry will promote the development of herbivorous animal husbandry and increase production and income of farmers and herdsmen. | http://www.moa.gov.cn/nybgb/2017/dlq/201712/t20171231_6133718.htm. |
| Guidance of Ministry of Agriculture on the adjustment of maize structure in sickle bend area | 2017 | Promote the integration of cultivation and breeding, implement grain to feed program in sickle bend area. Sickle bend area includes northeastern cold areas, northern agro-pastoral ecotone, northwestern dry aeolian sand region, the Taihang Mountain area and the southwest rocky desertification area. | http://www.moa.gov.cn/nybgb/2015/shiyiqi/201712/t20171219_6103893.htm |
| Promoting the dairy industry's revitalization and ensuring the quality and safety of dairy products | 2018 | It refers to generalize changing grain to forage program and develop silage corn, oat grass and other high quality forage industry, to improve the specialization of the forage, the scale of production, marketization, the yield of planting, the efficiency of cow production and the efficiency of the breeding. | http://www.gov.cn/zhengce/content/2018-06/11/content_5297839.htm |

**Supplementary Table 23 The forage production of non-competitive land in China**

| **Land type** | **Area (ha)** | **Forage** | **Production (Mt)** |
| --- | --- | --- | --- |
| Winter fallow cropland | 28,030,000 | Ryegrass | 487.5 |
| Summer fallow cropland | 1,474,667 | Green maize | 27.8 |
| Grain to forage cropland | 666,870 | Green maize | 7.3 |

**Supplementary Table 24** **Domestic demand, production and the gap for green maize under the scenarios.**

| # | Scenario | Domestic demand | Domestic production | Feed gap |
| --- | --- | --- | --- | --- |
|  |  |  |  |  |
|  |  |  |  |  |
|  |  | Mt | Mt | Mt |
| S0 | Business as usual | 26.5 | 36.3 | 0.0 |
| S1 | Green-source trade (meat & milk) | 26.5 | 36.3 | 0.0 |
| S2 | Green-source trade (feed) | 26.5 | 36.3 | 0.0 |
| S3 | Localized technology improvement | 37.5 | 36.3 | 1.2 |
| S4 | Green-source trade & technology | 37.5 | 36.3 | 1.2 |
| S5 | Total global supply | 0.0 | 36.3 | 0.0 |
| S6 | Total domestic supply | 47.7 | 36.3 | 11.4 |

**Supplementary Table 25** **Domestic demand, production and gap for grain maize under the scenarios.**

|  | Scenario | Domestic demand | Domestic production | Feed gap |
| --- | --- | --- | --- | --- |
|  |  |  |  |  |
|  |  |  |  |  |
|  |  | Mt | Mt | Mt |
| S0 | Business as usual | 53.8 | 50.9 | 2.9 |
| S1 | Green-source trade (meat & milk) | 53.8 | 50.9 | 2.9 |
| S2 | Green-source trade (feed) | 53.8 | 50.9 | 2.9 |
| S3 | Localized technology improvement | 69.5 | 50.9 | 18.5 |
| S4 | Green-source trade & technology | 69.5 | 50.9 | 18.5 |
| S5 | Total global supply | 0.0 | 50.9 | 0.0 |
| S6 | Total domestic supply | 95.7 | 50.9 | 44.7 |

**Supplementary Table 26** **Domestic demand, production and gap for soybeans under the scenarios.**

| # | Scenario | Domestic demand | Domestic production | Feed gap |
| --- | --- | --- | --- | --- |
|  |  |  |  |  |
|  |  |  |  |  |
|  |  | Mt | Mt | Mt |
| S0 | Business as usual | 18.6 | 21.7 | 0.0 |
| S1 | Green-source trade (meat & milk) | 18.6 | 21.7 | 0.0 |
| S2 | Green-source trade (feed) | 18.6 | 21.7 | 0.0 |
| S3 | Localized technology improvement | 22.9 | 21.7 | 1.2 |
| S4 | Green-source trade & technology | 22.9 | 21.7 | 1.2 |
| S5 | Total global supply | 0.0 | 21.7 | 0.0 |
| S6 | Total domestic supply | 32.6 | 21.7 | 10.9 |

**Supplementary Table 27 Domestic demand, production and gap for alfalfa under the scenarios.**

| # | Scenario | Domestic demand | Domestic production | Feed gap |
| --- | --- | --- | --- | --- |
|  |  |  |  |  |
|  |  |  |  |  |
|  |  | Mt | Mt | Mt |
| S0 | Business as usual | 29.8 | 68.6 | 0.0 |
| S1 | Green-source trade (meat & milk) | 29.8 | 68.6 | 0.0 |
| S2 | Green-source trade (feed) | 29.8 | 68.6 | 0.0 |
| S3 | Localized technology improvement | 39.8 | 68.6 | 0.0 |
| S4 | Green-source trade & technology | 39.8 | 68.6 | 0.0 |
| S5 | Total global supply | 0.0 | 68.6 | 0.0 |
| S6 | Total domestic supply | 61.1 | 68.6 | 0.0 |

**Supplementary Table 28** **Production and nutritional composition of main protein feed sources in China in 2014**

|  | Production (Mt) | DM  (%) | CP  (% DM) | CF  (% DM) | Ash  (% DM) | Ca  (% DM) | TP  (% DM) |
| --- | --- | --- | --- | --- | --- | --- | --- |
| Cottonseed meal | 5.2 | 89.6 | 36.3 | 6.4 | 6.9 | 0.3 | 0.9 |
| Rapeseed meal | 9.6 | 92.2 | 39.5 | 8.5 | 8.7 | 0.79 | 1.03 |
| Peanut meal | 7.3 | 89.0 | 55.2 | 8.1 | 6.4 | 0.34 | 0.33 |
| Wheat bran | 25.2 | 88.6 | 16.3 | 4.2 | 5.8 | 0.2 | 0.88 |

DM: dry matter; CP: crude protein; CF: crude fat; Ca: calcium; TP: total phosphorus.

**Supplementary Table 29** **Production and nutritional composition of main crop straws in China in 2014**

|  | Production  (Mt) | DM (%) | CP  (%DM) | CF  (%DM) | Ash  (%DM) | Ca  (%DM) | TP  (%DM) | NDF  (%DM) | ADF  (%DM) |
| --- | --- | --- | --- | --- | --- | --- | --- | --- | --- |
| Maize straw | 280.56 | 92.34 | 6.27 | 1.34 | 10.00 | 0.57 | 0.13 | 61.58 | 35.14 |
| Wheat straw | 157.77 | 93.71 | 3.69 | 0.88 | 8.37 | 0.32 | 0.07 | 73.18 | 45.39 |
| Rice straw | 201.99 | 92.47 | 4.34 | 1.50 | 12.04 | 0.43 | 0.13 | 61.75 | 36.95 |
| Soybean straw | 18.72 | 91.77 | 6.58 | 1.01 | 5.56 | 0.92 | 0.15 | 59.56 | 41.98 |

DM: dry matter; CP: crude protein; CF: crude fat; Ca: calcium; TP: total phosphorus; NDF: neutral detergent fiber; ADF: acid detergent fiber.

**Supplementary Table 30 Summary of uncertainty values assumed for the GHG estimation model parameters and data inputs used in the Monte Carlo analysis**

| **Parameter** | **Distribution** | **Uncertainty (1σ)** | **Reference** |
| --- | --- | --- | --- |
| CH_4_ emission factor for enteric fermentation | normal | ±50% | *54* |
| CH_4_ emission factor for manure management | normal | ±30% | *11* |
| N_2_O emission factor for chemical fertilizer production | normal | ±50% |  |
| CH_4_ emission factor for chemical fertilizer production | normal | ±50% |  |
| Direct emission factors for N_2_O from liquid manure | normal | ±80% | *54* |
| Direct emission factors for N_2_O from solid manure | normal | ±80% | *54* |
| Milk productivity | normal | ±10% |  |
| Cattle number | normal | ±10% | *12* |
| Chemical fertiliser application rate | normal | ±5% |  |
| Dry matter intake | normal | ±10% |  |
| Feed crop yield |  | ±5% |  |
| Cattle population structure | normal | ±10% |  |
| Direct emission factor for N_2_O emissions from fertilizer (or manure) application | normal | ±80% | *54* |
| Indirect N_2_O emission factor due to the volatilization of NH_3_ and NOx (fertiliser application) | normal | ±30% | *54* |
| Indirect N_2_O emission factor due to the volatilization of NH_3_ and NOx (manure used as fertiliser) | normal | ±40% | *54* |
| Electricity use on farm | normal | ±10% |  |
| Coal use on farm | normal | ±10% |  |
| Diesel use on farm | normal | ±10% |  |
| Animal excrement rate for urine | normal | ±25% | *21* |
| Animal excrement rate for faeces | normal | ±25% | *21* |
| Nitrogen content of urine | normal | ±0.25% | *21* |
| Nitrogen content of faeces | normal | ±0.25% | *21* |
| Animal mass of beef cattle | normal | ±10% |  |

**Supplementary Table 31 Summary of uncertainty values assumed for the NH_3_ estimation model parameters and data inputs used in the Monte Carlo analysis**

| **Parameter** | **Distribution** | **Uncertainty (1σ)** | **Reference** |
| --- | --- | --- | --- |
| NH_3_ emission factor for chemical fertilizer production | normal | ±50% |  |
| NH_3_ emission factor for manure application | normal | ±50% | *21* |
| NH_3_ emission factor for urine in housing | normal | ±50% | *21* |
| NH_3_ emission factor for faeces in housing | normal | ±50% | *21* |
| NH_3_ emission factor for solid manure in storage/treatment | normal | ±50% | *21* |
| NH_3_ emission factor for liquid manure in storage/treatment | normal | ±50% | *21* |
| Chemical fertiliser application rate | normal | ±25% | *55* |
| NH_3_ emission factor for chemical fertilizer application | normal | ±50% | *55* |
| Soil emission factor | normal | ±100% | *56* |
| Nitrogen fixation rate for nitrogen-fixing crop | normal | ±50% | *55* |
| NH_3_ emission factor for nitrogen-fixing crop | normal | ±50% | *55* |
| Animal excrement rate for urine | normal | ±25% | *55* |
| Animal excrement rate for faeces | normal | ±25% | *55* |
| Nitrogen content of urine | normal | ±0.25% | *55* |
| Nitrogen content of faeces | normal | ±0.25% | *55* |
| Percent of TAN | uniform | ±0.5% | *21* |
| Emission factor of traffic sources | normal | ±50% | *55* |
| Milk productivity | normal | ±10% |  |
| Animal mass of beef cattle | normal | ±10% |  |
| Feed crop yield | normal | ±5% | *57* |

**Supplementary Table 32** **GHG emissions intensities of beef meat and milk production in 2012 and 2050 for the major exporting nations.**

| **Exporting nation** | **Beef meat** | | **Milk** | |
| --- | --- | --- | --- | --- |
|  | (kg CO_2_ kg^-1^ carcass) | | (kg CO_2_ kg^-1^ milk) | |
|  | 2012 | 2050 | 2012 | 2050 |
| New Zealand | 12.1 | 8.1 | 0.8 | 0.7 |
| Australia | 9.9 | 8.1 | 1.1 | 0.6 |
| Uruguay | 18.4 | 13.1 | - | - |
| USA | - | - | 1.2 | 0.8 |

Note: “-” means that is not considered in this study.

**Supplementary Table 33 NH_3_ emissions intensities of beef meat and milk production in 2012 and 2050 for the major exporting nations**

| **Exporting nation** | **Beef meat** | | **Milk** | |
| --- | --- | --- | --- | --- |
|  | (kg NH_3_ t^-1^ carcass) | | (kg NH_3_ t^-1^ milk) | |
|  | 2012 | 2050 | 2012 | 2050 |
| New Zealand | 83.5 | 55.9 | 3.28 | 3.24 |
| Australia | 83.5 | 68.6 | 4.72 | 2.38 |
| Uruguay | 115 | 81.9 | - | - |
| USA | - | - | 8.6 | 1.81 |

**Supplementary Table 34 The GHG emissions intensities of feed production in 2012 and 2050 for the major exporting nations.**

|  | Soybean | | Maize | | Alfalfa | |
| --- | --- | --- | --- | --- | --- | --- |
|  | kg CO_2_ kg-1 product | | kg CO_2_ kg-1 product | | kg CO_2_ kg-1 product | |
|  | 2012 | 2050 | 2012 | 2050 | 2012 | 2050 |
| Brazil | 0.19 | 0.13 | - | - | - | - |
| USA | - | - | 0.23 | 0.16 | 0.62 | 0.43 |

**Supplementary Table 35 NH_3_ emissions intensities of feed production in 2012 and 2050 for the major exporting nations.**

|  | Soybean | | Maize | | Alfalfa | |
| --- | --- | --- | --- | --- | --- | --- |
|  | kg NH_3_ t^-1^ product | | kg NH_3_ t^-1^ product | | kg NH_3_ t^-1^ product | |
|  | 2012 | 2050 | 2012 | 2050 | 2012 | 2050 |
| Brazil | 0.4 | 0.3 | - | - | - | - |
| USA | - | - | 1.8 | 1.3 | 0.4 | 0.3 |

**Supplementary Table 36 List of nations in the six economic groups analyzed in this study using the income-dependent method** (modified from ref *4*). Nations were grouped into six groups (Group A-F) according to their ranking by per capita GDP (averaged from 2000 to 2007).

| Economic group^*^ | The 94 nations used as the basis for projections | % world population in 2009 (2050) |
| --- | --- | --- |
| A | Australia, Austria, Canada, Denmark, Finland, France, Germany, Ireland, Japan, Netherlands, Norway, Swaziland, Sweden, United Kingdom, United States of America | 11.1 (9.4) |
| B | Argentina, Chile, Greece, Israel, Italy, Republic of Korea, Malaysia, Mauritius, New Zealand, Portugal, Saudi Arabia, Spain, Trinidad and Tobago, Uruguay, Venezuela | 4.9 (4.3) |
| C | Botswana, Brazil, Colombia, Costa Rica, Ecuador, Guatemala, Iran, Jordan, Mexico, Thailand, Tunisia, Turkey, South Africa, China | 30 (23.3) |
| D | Algeria, Bolivia, Sri Lanka, Cuba, Dominican Republic, Egypt, El Salvador, Indonesia, Jamaica, Lebanon, Morocco, Paraguay, Peru, Philippines, Swaziland | 7 (6.9) |
| E | Bangladesh, Myanmar, Cameroon, Benin, Ghana, Honduras, India, Côte d'Ivoire, Mozambique, Nicaragua, Nigeria, Pakistan, Senegal, Viet Nam | 30.2 (33.7) |
| F | Central African Republic, Chad, Gambia, Guinea, Haiti, Kenya, Democratic People's Republic of Korea, Madagascar, Malawi, Mali, Nepal, Niger, Zimbabwe, Rwanda, Sierra Leone, Sudan (former), Togo, Uganda, Burkina Faso, Ethiopia, Zambia | 7.5 (12.2) |
| Total | All Groups A-F | 89.9 (90.8) |

**Supplementary Table 37 Country codes used in the main text.** Source: <https://countrycode.org>

| Country name | Country code | Country name | Country code |
| --- | --- | --- | --- |
| Algeria | DZ | Malaysia | MY |
| Argentina | AR | Mexico | MX |
| Australia | AU | Namibia | NA |
| Belarus | BY | Netherlands | NL |
| Brazil | BR | New Zealand | NZ |
| Canada | CA | Nicaragua | NI |
| Chile | CL | Pakistan | PK |
| China | CN | Paraguay | PY |
| Denmark | DK | Poland | PL |
| Egypt | EG | Republic of Korea | KR |
| Ethiopia | ET | Russian Federation | RU |
| EU | EU | Saudi Arabia | SA |
| EU28 | EU | Spain | ES |
| France | FR | Sweden | SE |
| Germany | DE | Switzerland | CH |
| India | IN | United Arab Emirates | AE |
| Indonesia | ID | United Kingdom | GB |
| Ireland | IE | Uruguay | UY |
| Italy | IT | USA | US |
| Japan | JP | Venezuela | VE |
| Jordan | JO | Viet Nam | VN |

**Supplementary Table 38 Overview of published studies of GHG emission intensities from ruminant production systems.**

| **Study** | **Location** | **System** | **Methodology and approach used** | **Functional unit** | **System boundary** | **Result** | **Unit** |  |
| --- | --- | --- | --- | --- | --- | --- | --- | --- |
| ***Beef*** |  |  |  |  |  |  |  |  |
| Peters et al., 2010^40^ | Australia | G | The methodology described by the National Greenhouse Gas Inventory Committee | the delivery of 1 kg hot standard carcass weight at the exit gate of the meat processing plant | All on-site and upstream processes at the farm, feedlot, and whole processing plant, including transport between these sites. | 12 | kg CO_2_-eq kg^-1^ HSCW |  |
| Cerri et al., 2016^41^ | Brazil | G | Adjusted tier 2 protocols from IPCC guidelines | 1 kg of carcass weight basis | Cradle to farm-gate | 9.02-15.5 | kg CO_2_-eq kg^-1^ carcass |  |
| Gonzalo et al., 2013 | New Zealand | G | Adjusted tier 2 protocols from IPCC guidelines |  | Cradle to farm-gate | 9 | kg CO_2_-eq kg^-1^ live weight |  |
| Peters et al., 2010^40^ | Australia | I | The methodology described by the National Greenhouse Gas Inventory Committee | the delivery of 1 kg hot standard carcass weight at the exit gate of the meat processing plant | All on-site and upstream processes at the farm, feedlot, and whole processing plant, including transport between these sites. | 9.9 | kg CO_2_-eq kg^-1^ HSCW |  |
| Pelletier et al., 2010^34^ | USA | I | ISO-compliant life cycle assessment | 1 kg of live-weight beef | Cradle to farm-gate | 15.5 | kg CO_2_-eq kg^-1^ live weight |  |
| ***Milk*** |  |  |  |  |  |  |  |  |
| Basset-Mens et al., 2009^42^ | New Zealand | G | IPCC methodology for New Zealand. | 1 kg milk | Direct on farm, purchased inputs and indirect nitrous oxide emissions. | 0.933 | kg CO_2_-eq kg^-1^ milk |  |
| Casey and Holden, 2005^43^ | Ireland | G | NA | 1 kg ECM | Cradle to farm-gate | 1.45 | kg CO_2_-eq kg^-1^ milk |  |
| Haas et al., 2001^44^ | Germany | G | NA | 1 kg milk | Cradle to farm-gate | 1.3 | kg CO_2_-eq kg^-1^ milk |  |
| Basset-Mens et al., 2009^42^ | New Zealand | I | IPCC methodology for New Zealand. | 1 kg milk | Direct on farm, purchased inputs and indirect nitrous oxide emissions. | 0.754 | kg CO_2_-eq kg^-1^ milk |  |
| Martin & Willaume, 2016^45^ | France | I | IPCC Tier 2 simplified method | 1 kg milk | Cradle to farm-gate | 1.01 | kg CO_2_-eq kg^-1^ milk |  |
| Gollnow et al., 2014^46^ | Australia | I | International Dairy Federation carbon footprint guidelines and the life cycle assessment standards ISO 14040 and 14044 | 1 kg FPCM | Cradle to farm-gate | 1.11 | kg CO_2_-eq kg^-1^ milk | |
| Flysjö et al., 2011^37^ | Sweden | I | NA | 1 kg ECM | Cradle to farm gate | 1.16 | kg CO_2_-eq kg^-1^ milk |  |
| Thoma et al., 2013^47^ | USA | I | NA | 1 kg FPCM | Cradle to farm gate | 1.23 | kg CO_2_-eq kg^-1^ milk |  |

Note: G indicates grazing system, and I indicates industrial system. ECM: energy corrected milk. FPCM: fat and protein corrected milk.

**Supplementary Table 39 Overview of published studies of NH_3_ emission intensities from ruminant production systems.**

| **Study** | **Location** | **System** | **Methodology and approach used** | **Functional unit** | **System boundary** | **Result** | **Unit** |
| --- | --- | --- | --- | --- | --- | --- | --- |
| ***Beef*** |  |  |  |  |  |  |  |
| Faulkner and Shaw, 2008^48^ | EU | G | NA | 1 kg meat |  | 129.8 | kg NH_3_ t^-1^ meat |
| Behera et al., 2013^49^ | New Zealand | G | NA | 1 kg meat | Manure management and application | 83.5 | kg NH_3_ t^-1^ meat |
| Behera et al., 2013^49^ | Australia | G | NA | 1 kg meat | Manure management and application | 83.5 | kg NH_3_ t^-1^ meat |
| Carew, 2010^50^ | Canada | I | Mass balance approach | NA | feeding to excretion, housing, manure storage and manure applied in the field | 126.0 | kg NH_3_ t^-1^ meat |
| Carew, 2010^50^ | USA | I | Mass balance approach | NA | feeding to excretion, housing, manure storage and manure applied in the field | 50.0 | kg NH_3_ t^-1^ meat |
| ***Milk*** |  |  |  |  |  |  |  |
| Thomassen et al., 2008^51^ | Australia | G |  |  |  |  |  |
| Jarvis and Ledgard, 2002^52^ | New Zealand | G | Mass balance approach | 1000 L milk | Grazed swards, cut swards, housing, farm manure, dirty water effluent, collection yard, laneways | 3.3 | kg NH_3_ t^-1^ milk |
| Jarvis and Ledgard, 2002^52^ | UK | I | Mass balance approach | 1000 L milk | Grazed swards, cut swards, housing, farm manure, dirty water effluent, collection yard, laneways | 9.4 | kg NH_3_ t^-1^ milk |
| Haas et al., 2001^44^ | Germany | I |  |  |  | 9.4 | kg NH_3_ t^-1^ milk |
| Thomassen et al., 2008^51^ | Netherlands | I | Life cycle assessment | 1 kg FPCM | Stable, pasture, storage and fertilizer application | 3.3 | kg NH_3_ t^-1^ FPCM |
| Cederberg and Flysjo , 2004^53^ | Sweden | I | Life cycle assessment | 1 kg ECM | Cradle to farm gate | 4.7 | kg NH_3_ t^-1^ ECM |

Note: G indicates grazing system, and I indicates industrial system. ECM: energy corrected milk. FPCM: fat and protein corrected milk.

**Supplementary Discussion**

**1 Cases study of ruminant livestock farms**

In China, ruminant production occurs in a wide range of systems from extensive grazing systems to intensive industrial systems. In grazing systems, most of the excretion is deposited directly on grasslands by livestock during the grazing period. When the livestock are kept in confinement, the solid part of the excretion is collected and removed but most of the liquid is discharged to the environment. In mixed systems, most of the solid excretion is collected and often applied to croplands, while the liquid fraction is only partially collected and the remainder is discharged to the wider environment. The farms we surveyed, such as those in Shandong, directly discharged liquid manure to the nearby river due to the high labour cost of collecting and transporting liquid manure. In industrial systems, because of limited land (or unpermitted land use), a large amount of the manure from industrial systems is discharged into surface waters or dumped into landfills with no treatment, for example in farms in Henan Province. Only a small part of the solid manure (after composting treatment) is transported to nearby land for growing vegetables and fruits. Although most of the animal waste in China is managed insufficiently, there are prospective “good news” stories of rapidly improving on-farm production practices in some regions. Here we present 10 case studies of advances in sustainable intensification of production practices in dairy farm and ruminant meat systems in China.

***Dairy farm case studies***

***Case study 1* Yijing dairy farm**, located in Shaoxing city (30.1 °N, 120.3 °E), Zhejiang Province, Southeast China. This area is one of the most developed areas in economic and social-ecological terms. The dairy farm is a representative example with the highest level of milk productivity in China with complete animal waste recycling in nearby croplands. The industrial feedlots are equipped with outer covers and internal machines, such as air conditioning, faecal cleaning machine, milking equipment, and so on. Milk productivity: 10.6 t head^-1^ yr^-1^, still lower than developed nations. Alfalfa hay is imported from the US. The animal wastes (both solid and liquid) from the feedlots are first collected for biogas production, and the biogas is used for electricity generation and for drying biogas residue. The dried biogas residue is finally used as bedding material in feedlots. The feedlots are coupled with the nearby feed croplands (Supplementary Figure 11). The biogas slurry, after aeration, is recycled to the nearby green maize croplands through a pipeline system. In winter when the green maize is harvested, cold season vegetables are planted for human consumption in the croplands.

***Case study 2* Xiandai dairy farm,** located in Bengbu (33.1 °N, 118.0 °E), Anhui Province, Southeast China. The milk productivity is 10.3 t head^-1^ yr^-1^. The management of animal waste is similar to that of Yijing dairy farm. The animal waste is used to produce biogas, 30% of the biogas is used for electricity generation, and the other 70% is used for boiler burning. The biogas slurry, after aeration, is returned to nearby alfalfa planting fields through a pipeline system.

***Case study 3* Binzhou dairy farm,** located in Binzhou (37.5 °N, 118.0 °E), Shandong Province, East China. The farm is composed of industrial feedlots, and milk productivity is 7.4 t head^-1^ yr^-1^. The animal waste reuse mode is different from the above case studies. Dairy cattle waste is sold to an earthworm breeding company. A variety of enzymes secreted from the digestive system of earthworms can decompose the organic matter in the manure rapidly (in 20 days). The earthworm castings are good organic fertilizer for flowers and greenhouse vegetables. The company sells the earthworms as Chinese medicine or bait.

***Case study 4* Xining dairy farm,** located in Xining (36.5 °N, 101.8 °E), Qinghai Province, Northwest China, the livestock waste is sold to the adjacent manure processing plant. Feed is supplied locally. The waste from the processing plant is separated into solid and liquid portions, and the solid part is further processed into organic fertilizer and then sold. The liquid part is used by the farmers to fertilize maize near the plant due to the difficulty and high cost of transporting the liquid manure.

***Case study 5*** **Mashan dairy farm,** located in Wuxi (31.5 °N, 120.1 °E), Jiangsu Province, Southeast China. The farm is composed of industrial feedlots, and milk productivity is 7.5 t head^-1^ yr^-1^. The alfalfa hay is imported from the US. This farm system is distinctive in that the biogas slurry is transported long distances by trucks to fertilize high-value horticultural export crops, such as peach trees.

***Case study 6* Dahengshan dairy farm,** located in Nanping (26.8 °N, 118.3 °E), Southeast China, Fujian Province. The farm is composed of industrial feedlots, and milk productivity is 8.0 t head^-1^ yr^-1^. This farm system is distinctive because the feedlot is coupled with constructed wetlands to treat the urine and wastewater.

***Beef cattle or sheep farm case studies***

***Case study 7* Jiaojiang beef farm,** located in Taizhou (28.6 °N, 121.5 °E), Zhejiang Province, Southeast China. The farm buys beef cattle from northern pastoral areas in autumn and fattens them for 3 to 4 months in Southeast China, where the air temperature in winter is suitable for beef cattle. The local corn and straw after harvest in autumn provides sufficient feedstuff for beef cattle fattening. The temperature in summer in Zhejiang province is too high for beef cattle fattening. So this breeding mode takes advantage of the different climatic zones across China.

***Case study 8* Yilanchun beef company,** located in Dingxi (35.5 °N, 104.6°E), Gansu Province, Northwest China. The beef cattle faeces and urine is used to produce biogas and the biogas slurry is recycled to grow vegetables.

***Case study 9* Huocheng sheep farm,** located in Huocheng (44.1 °N, 80.8 °E), Xinjiang Province, Northwest China. In this feedlot system, the urine is not managed but is discharged directly to the ground where it evaporates rapidly due to the dry climate. The solid manure is cleaned only once before the sheep are slaughtered. The solid manure is used by nearby farmers to fertilize crops.

***Case study 10* Chengtangcun** **sheep farm,** located in Huizhou (29.9 °N, 118.3 °E), Anhui Province, Southeast China. In Huizhou the climate is warm and humid, and the animal waste must be cleared regularly to prevent disease. The floor of the sheep feedlot is perforated, and urine drops through the floor into a tank below, while the solid manure on the feedlot floor is collected every day and applied to croplands.

**2 Land competitions between food and feed in China**

The land use competition between grain crops and livestock feed can occur in response to the continuously increasing livestock breeding. Fortunately, some favorable factors can avoid such competition in China, mainly including two aspects. First, competition could be relieved by the seasonal fallow croplands. In recent years, fallow croplands have mainly been used to produce feed crops, which can reduce competition for cropland.

*Summer fallow croplands*: In the temperate region of northern China, the main grain crop is winter wheat, which is harvested in early June. Then there is nearly 100 days before sowing next season wheat in October. In this period, the rainfall and temperature conditions are suitable to the growth of plants with short growing period, but not suitable for an extra grain crop rotation. This kind of cropland is the summer fallow cropland, which is no use in past decades for a few ruminant livestock farms. Now many areas have been shifting to green maize croplands to meet demand of dairy farms. There is a large area (1,474,667 ha) of summer fallow croplands (Supplementary Table 23), which have about 100 days after the harvest of wheat in late May and before winter wheat sowing in the following year, mainly in Shanxi, and Shaanxi Province of North China. Planting green maize on summer fallow fields could allow for seasonal rotation between forage production and agricultural production.

*Winter fallow croplands*: In the subtropical climate regions of central-southern China, the croplands have a fallow period of about 110 days from November when the crops are harvested in until March for sowing next crops. In the fallow period, the light and temperature are not sufficient but plants can grow slowly, while water resource and labour in this period are sufficient^72^. This kind of cropland used to be the winter fallow cropland in the past years due to there is a few ruminant livestock farms. Recently, many areas of the winter fallow cropland have been converted to planting ryegrass^73^ to meet the increasing demands for forages from ruminant livestock farms. The winter fallow croplands are typically used for ryegrass (*Lolium* sp.) production.

Utilizing winter or summer fallow croplands to plant forage crops would reduce direct competition with food croplands. Such multiple cropping systems have existed in China for thousands of years.

Second, in 2015, the China’s Central Government has promulgated a new program, i.e. changing land use for grain to forage cultivation (grain to forage). In China’s cold northeastern areas, northern agro-pastoral ecotone, northwestern dry aeolian sand region, the Taihang Mountain area and the southwest rocky desertification area (Supplementary Table 22), the grain maize yield is low and inconsistent due to insufficient and variable temperature or water conditions. Then the China’s Central Government decided to change grain maize to forage and feed planting (e.g. green maize and soybeans) in these areas. The total area that h implemented this program is approximately 666,870 ha (Supplementary Table 23). The program of grain to forage has been adjusting the land use pattern and optimizing the planting structure in China, with the aim of increasing forage production.

In sum, these types of approaches have a high potential to mitigate competition for land between food crops and feed crops.

Different from the forage, the gap in some grain supply could not be fully closed by domestic cropland, even technological improvement, so it needs to import from other nations. We took land use for feed and forage cultivation as exogenous variables to discuss potential extra land demand in the future. First, we calculated the demand for forage and feed (green maize, maize, soybean, and alfalfa) in all of our six model scenarios. Second, we calculated the potential for forage production based on current land area of non-competitive cropland and grain to forage cropland in China (see Supplementary Table 23). Third, we evaluated the gap between demand and supply. From this analysis, we find that local cropland areas can meet the demand for feed and forage in 2050, indicating that it is unnecessary to convert forest to cropland for feed crop production.

**(a) *Green maize***

***i. Demand for green maize*** Based on our models, the demand for green maize in 2050 will be 26.5 to 47.7 Mt across the six scenarios (Supplementary Table 24).

***ii. Potential for green maize production in China*** Summer fallow cropland: summer fallow croplands in China^71^ can potentially produce 27.8 Mt green maize per annum. It is worth noting that the production of green maize in summer fallow cropland will not significantly increase GHG emissions caused by land use change. Although fertilizer is applied in fallow croplands for production and this incurs extra GHG and ammonia emissions, some studies report that planting forage on fallow land can increase soil organic matter, which contributes to GHG mitigation^74^. We have included emissions in our calculations.

Grain to forage policy: In 2015, the Chinese government clearly promoted the grain to forage, i.e. changing grain maize to silage corn or soybean or other forage in China’s northeastern cold areas, northern agro-pastoral ecotone, northwestern dry aeolian sand region, the Taihang Mountain area and the southwest rocky desertification area. The cropland under the grain to forage program can potentially provide 7.29 Mt green maize per annum.

In sum, the green maize planted on the non-competitive land and grain to forage land is estimated to be as high as 35.09 Mt. Considering the existing production of green maize, the total production of green maize could reach 36.3Mt in China each year.

***iii.* *Balance of green maize*** The existed studies report that green maize silage is beneficial in improving milk productivity^75^, so we give priority to the demand of dairy cows for green maize (Supplementary Table 24). The results show that the gap of green maize is 1.2 Mt, 1.2 Mt and 11.4 Mt in scenarios S3, S4, and S6, respectively.

In addition, ryegrass production in winter fallow cropland can reach 487.5 Mt, which could potentially fill the gap in green maize availability. We conclude that the demand and supply can be balanced if croplands are used fully and efficiently, and it is unnecessary to convert forest to cropland. In fact, in China, it is impractical to convert forest or grassland to agricultural land. To restore degraded ecosystems, the central Government has initiated several national ecological restoration projects since the late 1990s, implementing stringent protection regulations in arable lands and natural ecosystems^76,77^, such as the Natural Forest Protection Program (Supplementary Table 22).

**(b) *Grain maize***

***i. Demand for grain maize*** Based on our models, the demand for grain maize in the ruminant livestock sector in 2050 will be 53.8 to 95.7 Mt across the six scenarios (Supplementary Table 25).

***ii. Potential for grain maize production in China*** Based on trends in China’s maize production from 1961 to 2014, we calculated that future production will reach 514.9 Mt, which is over 2.2 times higher than that in 2016 (231.8 Mt). This increase in maize production could be achieved by closing the yield gap instead of area expansion^78^. Currently, the average yield of maize in China is 5.9 t ha^-1^ yr^-1^, which is only 50% of that in USA (11.0 t ha^-1^ yr^-1^).

***iii.* *Balance of grain maize*** In China, nearly 70% of domestic maize production is used as feed^79^ and 14.1% of them are consumed by ruminant livestock^81^. We assumed that these ratios keep constant to 2050 and estimated that there will be 50.9 Mt grain maize for ruminant livestock in 2050, which will cause small gaps in scenarios. These small gaps can be filled by China’s large grain maize stock (250 Mt) and import.

**(c) *Soybean***

***i. Demand for soybean*** Soybean cake is the by-product of soybean processing. In our calculation, we have used soybean cake to calculate soybean demand. Based on our models, the demand for soybean in the ruminant livestock sector in 2050 will be 18.6 to 32.6 Mt across the six scenarios (Supplementary Table 26).

***ii. Potential for soybean production in China*** Based on the trend in China’s soybean production from 1961 to 2014, we calculated that the production of soybean will reach 21.7 Mt in 2050, which is nearly double that in 2016 (11.97 Mt). The increase in soybean production can also be achieved by closing the yield gap instead of area expansion^78^. Currently, the average yield of soybean in China is 1.8 t ha^-1^ yr^-1^, which is only 50% of that in the USA (3.5 t ha^-1^ yr^-1^). Soybean cake or soybean meal processed from soybean is the protein feed of ruminant livestock. It is notable that soybean production in China will increase in the future, as China’s Ministry of Agriculture recently published guidance on promoting the development of soybean production (<http://www.moa.gov.cn/govpublic/ZZYGLS/201604/t20160412_5091357.htm>).

***iii.* *Balance of soybean*** Although there are gaps between supply and demand by 2050, there are many kinds of protein feed that can supplement soybean cake, such as cottonseed meal, rapeseed meal, peanut meal and wheat bran (Supplementary Table 28). The production of meal with high crude protein content (cottonseed meal, rapeseed meal, peanut meal) reached 22 Mt in 2014, which can fill the gap in soybean demand in each of the six scenarios.

**(d) *Alfalfa***

***i. Demand for alfalfa*** Based on our models, the demand for alfalfa in the ruminant livestock sector in 2050 will be 29.8 to 61.1 Mt across the six scenarios (Supplementary Table 27).

***ii. Potential of alfalfa production*** Based on the trend in China’s alfalfa production from 2001 to 2015, we calculated that alfalfa production will reach 68.6 Mt in 2050. We also calculated alfalfa production in 2020 (38.2 Mt), which was in accordance with the planning production (35.8 Mt) in China’s National Alfalfa Industry Development Plan published (2016-2020) by the Ministry of Agriculture. The plan will increase policy support and guidance, strengthen the construction of an alfalfa grass breeding base, vigorously develop high yield and high quality alfalfa planting and processing. Hence, it is highly likely that production and yield improvement of alfalfa will be achieved.

***iii.* *Balance of alfalfa*** We calculated that alfalfa production will reach 68.6 Mt in 2050, which can fill the gap in alfalfa demand under all six scenarios.

**(e) *Crop straw***

In addition, crop straw is the most common roughage for livestock feed, which is rich in nutritional composition (Supplementary Table 29), and plays an important role in ruminant livestock breeding. As a non-competitive feed resource, straw is not only cheap but also has a certain feeding value from its own nutritional components and rumen degradation characteristics^80^. We calculated that the amount of straw from corn, wheat, rice and soybeans accounted for 67% of the total crop straw in China (Supplementary Table 29). These straws can be used as roughage for beef cattle. The efficient utilization of straw for fodder not only expands the source of feed, promotes a large increase in the production of livestock and livestock industry, saves food, guarantees the national food security, increases employment opportunities, and promotes the increase of farmers' income; it can also improve the environment and promote more sustainable development of agriculture.

**Supplementary References**

1. ISO. Environmental Management. The ISO 14,000 family of international standards, ISO Standards Collection on CD-ROM, Geneve (2006).
2. Cederberg, C. & Mattsson, B. Life cycle assessment of milk production — a comparison of conventional and organic farming. *J. Clean Prod.* **8**, 49-60 (2000).
3. FAO, Food and Agriculture Organization, Statistics Database (2017); <http://faostat.fao.org/>
4. Tilman, D. et al. Global food demand and the sustainable intensification of agriculture. *Proc. Natl. Acad. Sci. USA* **108**, 20260-20264 (2011).
5. Tilman, D., & Clark, M. Global diets link environmental sustainability and human health. *Nature* **515**, 518-522 (2014).
6. Alexandratos, N., & Bruinsma, J. *World Agriculture Towards 2030/2050: The 2012 Revision* (FAO, Rome, 2012).
7. Ward, K. The world in 2050. *Global Economics, HSBC, Global Research* 1–40 (2012) (11 January).
8. Meyer, I. et al. Scenarios for regional passenger car fleets and their CO_2_ emissions. *Energ. Pol.* **41**, 66–74 (2012).
9. Hawksworth, J. The world in 2050: How big will the major emerging market economies get and how can the OECD compete. (2006).
10. Zhang, W. et al. New technologies reduce greenhouse gas emissions from nitrogenous fertilizer in China. *Proc. Natl. Acad. Sci. U. S. A.* **110**, 8375-8380 (2013).
11. IPCC, Guidelines for national greenhouse gas inventories. *(IPCC WGI technical support unit, Bracknell)*, (2006).
12. National Coordination Committee on Climate Change (NCCC). The people’s republic of China national greenhouse gas inventory. *China Environmental Science Press* (2007).
13. Tian, S. X. Estimation of greenhouse gases inventory of agriculture in Inner Mongolia. *Inner Mongolia University. Master thesis* (2017) (in Chinese with English abstract)
14. Wang, X. et al. Environmental impacts and resource use of milk production on the North China plain, based on life cycle assessment. *Sci. Total Environ.* **625**, 486-495 (2018).
15. Wells, C. Total Energy Indicators of Agricultural Sustainability: Dairy Farming Case Study. *Wellington: Ministry of Agriculture and Forestry* (2001).
16. Huang, W.Q. Carbon footprint assessment methodology of milk production in intensive dairy farm and case study. *Chinese Academy of Agricultural Sciences. Master thesis.* (2015) (in Chinese with English abstract).
17. Ma, Z. H., Wang, M. Z., Ding, L. Y. & Liu, J. J. Emissions of greenhouse gases from an industrial beef feedlot farm as evaluated by a life-cycle assessment method. *J. Agro-Environ. Sci.* **29**, 2244 - 2252 (2010). (in Chinese with English abstract)
18. Zhou, J.Y. Analysis on environmental impact of livestock and poultry breeding based on life cycle assessment. *Qingdao University of Science & Technology. Master thesis* (2017) (in Chinese with English abstract)
19. Ding, N., Yang, J.X., Lu, B. Life cycle inventory analysis of provincial thermal electricity in China. *Acta Ecologica Sinaca* **36**, 7192-7201 (2016). (in Chinese with English abstract)
20. Ding, N., Yang, J.X. Life cycle inventory analysis of fossil energy in China. *China Environmental Science* **35**, 1592-1600 (2015). (in Chinese with English abstract)
21. Huang, X. et al. A high-resolution ammonia emission inventory in China. *Glob. Biogeochem. Cycle* **26**, 239-256 (2012).
22. Salazar, F. et al. Ammonia emission from a permanent grassland on volcanic soil after the treatment with dairy slurry and urea. *Atmos. Environ.* **95**, 591-597 (2014).
23. Ma, L. et al. Modeling nutrient flows in the food chain of China. *J. Environ. Qual.* **39**, 1279-1289 (2010).
24. Chapagain, A. K.; Hoekstra, A. Y. Water footprints of nations. Volume 2: Appendices. Value of Water Research Series 2004, No. 16.
25. IPCC *Climate Change 2014: Synthesis Report* (Contribution of Working Groups I, II and III to the Fifth Assessment Report of the Intergovernmental Panel on Climate Change). In: Core Writing Team, Pachauri, R.K., Meyer, L.A., (Eds.). IPCC, Geneva, Switzerland.
26. Goedkoop, M. J. et al. ReCiPe 2008, A life cycle impact assessment method which comprises harmonised category indicators at the midpoint and the endpoint level. National Institute for Public Health and the Environment, Bilthoven, Netherlands. (2009).
27. Weidema, B. P. Using the budget constraint to monetarise impact assessment results. *Ecol. Econ.* **68**, 1591-1598 (2009).
28. Grassini, P. & Cassman, K. G. High-yield maize with large net energy yield and small global warming intensity. *Proc. Natl Acad. Sci. USA*. **109,** 1074-1079 (2012).
29. Nielsen, P. H., Nielsen, A. M., Weidema, B. P., Dalgaard, R., Halberg, N. LCA food data base. Available from: [www.lcafood.dk/database](http://www.lcafood.dk/database) (2013).
30. Duhl, T. R., Gochis, D., Guenther, A., Ferrenberg, S. & Pendall, E. Nitrogen food-print: N use related to meat and dairy consumption in France. *Biogeosciences* **10**, 471-481 (2013).
31. Dalgaard, R. et al. LCA of soybean meal. *Int. J. Life Cycle Ass.* **13**, 240-254 (2008).
32. Bouwman, A. F. et al. A global high‐resolution emission inventory for ammonia. *Global Biogeochem. Cycles* **11**, 561-587 (1997).
33. Peters, G. M. et al. Red meat production in Australia: life cycle assessment and comparison with overseas studies. *Environ. Sci. Technol.* **44**, 1327-1332 (2010).
34. Pelletier, N, Pirog, R, Rasmussen, R. Comparative life cycle environmental impacts of three beef production strategies in the Upper Midwestern United States. *Agric. Syst.* **103**, 380-389 (2010).
35. Eshel, G., Shepon, A., Makov, T. & Milo, R. Land, irrigation water, greenhouse gas, and reactive nitrogen burdens of meat, eggs, and dairy production in the United States. *Proc. Natl. Acad. Sci. U. S. A.* **111**, 11996-12001 (2014).
36. Gollnow, S. et al. Carbon footprint of milk production from dairy cows in Australia. *Int. Dairy J.* **37**, 31-38 (2014).
37. Flysjö, A., Cederberg, C., Henriksson, M. & Ledgard, S. How does co-product handling affect the carbon footprint of milk? Case study of milk production in New Zealand and Sweden. *Int. J. Life Cycle Ass.* **16**, 420-430 (2011).
38. Bjorneberg, D. L. et al. Measurement of atmospheric ammonia, methane, and nitrous oxide at a concentrated dairy production facility in Southern Idaho using open-path FTIR spectrometry. **52**, 1749-1756 (2009).
39. Thomassen, M. A., Calker, K. J. V., Smits, M. C. J., Iepema, G. L. & Boer, I. J. M. D. Life cycle assessment of conventional and organic milk production in the Netherlands. *Agr. Syst.* **96**, 95-107 (2008).
40. Peters, G. M., Rowley, H. V., Wiedemann, S., Tucker, R., Short, M. D., & Schulz, M. (2010). Red meat production in Australia: life cycle assessment and comparison with overseas studies. *Environ. Sci. Technol.* **44,** 1327-32 (2010).
41. Cerri, C. C, Moreira, C. S, Alves, P. A et al. Assessing the carbon footprint of beef cattle in Brazil: a case study with 22 farms in the State of Mato Grosso. *J. Clean Prod.* **112**, 2593-2600 (2016).
42. Basset-Mens, C., Ledgard, S., Boyes, M. Eco-efficiency of intensification scenarios for milk production in New Zealand. *Ecol. Econ.* **68**, 1615–1625 (2009).
43. Casey, J.W., Holden, N.M., Analysis of greenhouse gas emissions from the average Irish milk production system. *Agric. Syst.* **86,** 97–114 (2005).
44. Haas, G., Wetterich, F., Köpke, U. Comparing intensive, extensified and organic grassland farming in southern Germany by process life cycle assessment. *Agric. Ecosyst. Environ.* **83,** 43–53 (2001).
45. Martin, G , Willaume, M. A. diachronic study of greenhouse gas emissions of French dairy farms according to adaptation pathways. *Agric. Ecosyst. Environ.* **221**, 50-59 (2016).
46. Gollnow, S. et al. Carbon footprint of milk production from dairy cows in Australia. *Int. Dairy. J.* **37**, 31-38 (2014).
47. Thoma, G., Popp, J., Shonnard, D., Nutter, D., Matlock, M., & Ulrich, R. et al. Regional analysis of greenhouse gas emissions from USA dairy farms: a cradle to farm-gate assessment of the American dairy industry circa 2008. *Int. Dairy J.* **31**, S29-S40 (2013).
48. Faulkner, W. B., & Shaw, B. W. Review of ammonia emission factors for United States animal agriculture. *Atmos. Environ.* **42**, 6567-6574 (2008).
49. Behera, S. N. et al. Ammonia in the atmosphere: a review on emission sources, atmospheric chemistry and deposition on terrestrial bodies. *Environ. Sci. Pollut. Res.* **20**, 8092-8131 (2013).
50. Carew, R. Ammonia emissions from livestock industries in Canada: feasibility of abatement strategies. *Environmental pollution* (*Barking, Essex : 1987*) **158**, 2618 (2010).
51. Thomassen, M. A. et al. Life cycle assessment of conventional and organic milk production in the Netherlands. *Agric. Syst*. **96**, 95-107 (2008).
52. Jarvis, S. C., & Ledgard, S. Ammonia emissions from intensive dairying: a comparison of contrasting systems in the united kingdom and New Zealand. *Agric. Ecosyst. Environ.* **92**, 83-92 (2002).
53. Cederberg, C., & Mattsson, B. Life cycle assessment of milk production — a comparison of conventional and organic farming. *J. Clean Prod.* **8**, 49-60 (2000).
54. Monni, S., Syri, S., Savolainen, I., Uncertainties in the Finnish greenhouse gas emission inventory. *Environ. Sci. Policy* **7,** 87-98 (2004).
55. Beusen, A. H. W. et al. Bottom-up uncertainty estimates of global ammonia emissions from global agricultural production systems. *Atmos. Environ*. **42**, 6067-6077 (2008).
56. Olivier, J.G. J. et al. Global air emission inventories for anthropogenic sources of NOx, NH3 and N2O in 1990. *Environ. Pollut*. **102**, 135-148 (1998).
57. Zhao, Y. et al. Quantifying the uncertainties of a bottom-up emission inventory of anthropogenic atmospheric pollutants in China. *Atmos. Chem. Phys*. **11**, 2295-2308 (2011).
58. Gerber, P. J. et al. Tackling Climate Change through Livestock – A Global Assessment of Emissions and Mitigation Opportunities. *Food and Agriculture Organization of the United Nations (FAO)* Rome (2013).
59. Webb, J. et al. Managing ammonia emissions from livestock production in Europe. *Environ. Pollut.* **135**, 399-406 (2005).
60. Wagner, S., Angenendt, E., Beletskaya, O. & Zeddies, J. Costs and benefits of ammonia and particulate matter abatement in German agriculture including interactions with greenhouse gas emissions. *Agr. Syst.* **141**, 58-68 (2015).
61. Hou, Y. et al. Nutrient recovery and emissions of ammonia, nitrous oxide, and methane from animal manure in Europe: effects of manure treatment technologies. *Environ. Sci. Technol*. **51** (2017).
62. Foged, H. L. et al. Inventory of manure processing activities in Europe. *Recercat Principal* (2012).
63. SDPCCPD (State Development Planning Commission of China Price Department). *China Agricultural Cost and Benefit Data Compilation*. China Statistics Press, Beijing (2012). (in Chinese)
64. Gao, X. P. et al. Effects of mixed fertilizer applications on hay yield of alfalfa. *Journal of Anhui Agri. Sci.* **2011,** 39(8), 4753-4755. (in Chinese with English abstract)
65. Li, R. X. The influence of different fertilization levels on yields, nutrient absorption and soil fertility of alfalfa. *Xinjiang Agricultural University* (2007). (in Chinese with English abstract)
66. Hao, M. D., Zhang, C. X., Wei, X. R., Wang, X. G., Gao, C. Q. Effects of rotation and fertilization to alfalfa productivity on the Loess Plateau. *Acta Agrectir Sinica.* **3**, 195-198. (2004). (in Chinese with English abstract)
67. Fan, F. et al. Effects of fertilization on yield and nutrient content of Aohan alfalfa. *Chinese Journal of Grassland* **5**, 36-42. (2007) (in Chinese with English abstract)
68. Pan, L. et al. Effects of fertilizers and sowing rates on growth characteristics and forage yields of alfalfa in Yangzhou region. *Acta Agrectir Sinica* **6**: 1099-1104 (2012). (in Chinese with English abstract)
69. Zhou, Y., Wang, S. P., Chen, M. J. Effects of sulfur fertilizer on Medicago quality and digestibility in the Beijing region. *Acta Agrectir Sinica* **6**, 67-72. (2005) (in Chinese with English abstract)
70. Xu, S. L. et al. Studying influence of different fertilizer treatments on alfalfa growth characteristic. *Grass and animal husbandry* 23-25 (2009). (in Chinese with English abstract)
71. MOA (Ministry of Agriculture of the People’s Republic of China). China grass statistics 2015 (2016) (in Chinese).
72. Liu L, Xu X, Hu Y, *et al*. Efficiency analysis of bioenergy potential on winter fallow fields: A case study of rape. *Sci. Total Environ.* **628–629**, 103-109 (2018).
73. Qiao, W. Y., Gu, H. R., Shen, Y. X. Effects of planting Italian ryegrass in winter fallow fields on soil fertility and microorganisms. *Pratacultural Science*. **34**, 240-245 (2017) (in Chinese with English abstract).
74. Wang, L. H. Effects of former stubble winter cover crops on microbial characteristics in paddy soil. *Journal of Soil and Water Conservation*. **21**, 164-167 (2007).
75. Bal, M. A., Shaver, R. D., Jirovec, A. G., Shinners, K. J., & Coors, J. G. Crop processing and chop length of corn silage: effects on intake, digestion, and milk production by dairy cows. *J. Dairy Sci.* **83**, 1264-1273 (2000).
76. Fang, J. Y. et al. Climate change, human impacts, and carbon sequestration in China. *Proc. Natl. Acad. Sci. U. S. A.* **115**, 4015-4020 (2018).
77. Bryan, B. A. et al. China’s response to a national land-system sustainability emergency. *Nature* **559**, 193-204 (2018).
78. Mueller, N. D. et al. Closing yield gap through nutrient and water management. *Nature* **490**, 254-257 (2012).
79. FAO, Food and Agriculture Organization, Statistics Database; <http://faostat.fao.org/> (2018).
80. Wang, H. M. et al. Chinese crop straw resource and its utilization status. *Science & Technology Review*. **35**, 81-88 (2017) (in Chinese with English abstract).
81. Bai, Z. H. et al. Nitrogen and phosphorus use efficiencies in dairy production in China. *J. Environ. Qual.* **42**, 990-1001 (2013).
82. Havlík, P. et al. Climate change mitigation through livestock system transitions. *Proc. Natl Acad. Sci. USA* **111**, 3709-3714 (2014).
